# Identification of Bacterial Oligopeptidase B Inhibitors from Microbial Natural Products: Molecular Insights, Docking Studies, MD Simulations, and ADMET Predictions

**DOI:** 10.3390/ph18050709

**Published:** 2025-05-11

**Authors:** Malik Suliman Mohamed, Tilal Elsaman, Magdi Awadalla Mohamed, Eyman Mohamed Eltayib, Abualgasim Elgaili Abdalla, Mona Timan Idriss

**Affiliations:** 1 Department of Pharmaceutics, College of Pharmacy, Jouf University, Sakaka 72388, Saudi Arabia; emeahmed@ju.edu.sa; 2 Department of Pharmaceutical Chemistry, College of Pharmacy, Jouf University, Sakaka 72388, Saudi Arabia; maelhussein@ju.edu.sa; 3 Department of Clinical Laboratory Sciences, College of Applied Medical Sciences, Jouf University, Sakaka 72388, Saudi Arabia; aealseddig@ju.edu.sa; 4 Department of Nursing College, Northern Private College of Nursing, Arar 73312, Saudi Arabia; mona@nec.edu.sa

**Keywords:** oligopeptidase B, *Serratia marcescens*, *Stenotrophomonas maltophilia*, antimicrobial agents, in silico drug discovery, structure-based drug discovery

## Abstract

**Background/Objectives:** The increasing threat of antibiotic resistance and the declining efficiency of traditional drug discovery pipelines highlight the urgent need for novel drug targets and effective enzyme inhibitors against infectious diseases. Oligopeptidase B (OPB), a serine protease with trypsin-like specificity that processes low-molecular-weight peptides and oligopeptides, is present in bacteria and certain parasites but absent in mammals. This unique distribution makes OPB an attractive and selective target for antimicrobial drug development. **Methods:** Three-dimensional models of OPB from *Serratia marcescens* and *Stenotrophomonas maltophilia*, previously identified by our research group, were constructed via homology modeling using the best available OPB template from the RCSB Protein Data Bank. The *S. marcescens* OPB model was subjected to high-throughput virtual screening (HTVS) against the Natural Products Atlas (npatlas) database. Top-ranking compounds were further evaluated using Glide standard precision (SP) and extra precision (XP) docking protocols. Binding affinities were refined using molecular mechanics with generalized born and surface area (MM–GBSA) calculations. Molecular dynamics (MD) simulations assessed binding stability, while absorption distribution metabolism excretion and toxicity (ADMET) profiling evaluated drug-likeness and pharmacokinetic properties. **Results:** Ten natural product compounds demonstrated stronger binding affinities than antipain, a well-known oligopeptide-based protease inhibitor, as indicated by their more favorable MM–GBSA scores of −60.90 kcal/mol (*S. marcescens*) and −27.07 kcal/mol (*S. maltophilia*). Among these, dichrysobactin and validamycin E consistently exhibited favorable binding profiles across both OPB models. MD simulations confirmed the stability of their interactions with OPB active sites, maintaining favorable binding conformations throughout the simulation period. ADMET analysis suggested that while both compounds show promise, lead optimization is required to enhance their drug-like characteristics. **Conclusions:** This study identifies dichrysobactin and validamycin E as promising OPB inhibitors with potential antimicrobial activity. These findings support their further development as selective and potent agents against bacterial pathogens, including resistant strains, and underscore the need for experimental validation to confirm their efficacy and safety.

## 1. Introduction

Infectious diseases have garnered significant attention across all sectors of society due to their substantial impact on global health. As leading causes of morbidity and mortality, they affect individuals across diverse socioeconomic backgrounds and pose serious challenges to public health systems, social stability, and economic growth [1]. Forecasts suggest that without strategic interventions, the burden of infectious diseases may continue to rise globally, further straining healthcare resources and widening health disparities [2]. Several factors drive the ongoing global health crisis in infectious diseases, including the surge in antibiotic resistance, the overuse of broad-spectrum drugs, gaps in diagnostics and vaccines, and the emergence or resurgence of pathogenic microbes. The reliance on empirical treatments has hindered advancements in specialized diagnostics while inadvertently accelerating resistance and complicating antimicrobial therapies [3]. The global health system continues to combat infectious diseases, yet the ongoing emergence and resurgence of these illnesses, coupled with evolving resistance mechanisms, pose significant challenges. These issues undermine progress toward sustainable development goals and compromise the effectiveness of treatments, resulting in prolonged illness, increased disability, and higher mortality rates [4]. In addition, recent evidence underscores the critical need for stronger public health emergency preparedness, including timely surveillance, coordinated response frameworks, and resilient healthcare infrastructures to effectively manage infectious disease outbreaks and mitigate their long-term impacts [5].

Although at least 1513 bacterial pathogens capable of infecting humans were identified before 2021, the rapid emergence of new bacterial threats remains a growing concern. This underscores the urgent need for proactive control measures to combat these deadly pathogens [6]. Among them, Gram-negative bacteria pose a particularly serious challenge in healthcare due to their intrinsic and acquired resistance mechanisms, including horizontal gene transfer, efflux pumps, and other adaptive strategies. Their reduced susceptibility to antibiotics makes them a major public health threat, especially in hospital settings, where they cause severe infections, often requiring intensive care. These pathogens contribute to high morbidity and mortality, further complicating treatment efforts and straining healthcare systems [7]. The number of deaths associated with resistance to a single class of antibiotics, carbapenems, among Gram-negative bacteria has sharply increased from 619,000 in 1990 to 1.03 million in 2021. Furthermore, projections suggest that without additional policy interventions, global deaths attributable to antimicrobial resistance (AMR) could reach 39 million, while those associated with AMR could rise to 169 million between 2025 and 2050, equating to thirteen fatalities per minute [8]. This alarming trend underscores the urgent need for accelerated drug discovery programs and effective control measures to combat these deadly pathogens.

*Serratia marcescens* is an opportunistic pathogen responsible for numerous outbreaks and healthcare-associated infections. It is particularly concerning due to its ability to develop resistance to multiple antibiotics. This emerging bacterium exhibits significant genetic adaptability, allowing it to survive and persist in diverse environments. It has been isolated from various medical equipment, including surgical and oxygenation devices, syringes, and fomites, as well as from patients and healthcare personnel [9]. One of the major challenges in treating *S. marcescens* infections is its high level of antibiotic resistance, which results from a combination of intrinsic, acquired, and adaptive mechanisms. This resistance limits available treatment options and contributes to prolonged hospital stays for infected patients, increasing both healthcare costs and the risk of further complications [10]. Moreover, multidrug-resistant *S. marcescens* exhibits complex adaptive responses to antibiotic pressure, enhancing its survival and persistence in hostile clinical environments [11]. Given its clinical significance and resistance profile, *S. marcescens* is a prime target for research focused on identifying novel inhibitors with unique mechanisms of action. The discovery of such agents could not only aid in controlling this pathogen but also offer broad-spectrum solutions against other resistant bacterial species. Similar to *S. marcescens*, *Stenotrophomonas maltophilia* has emerged as a multidrug-resistant opportunistic pathogen with global significance. *S. maltophilia* is increasingly recognized as a high-risk pathogen in critically ill patients, necessitating prompt diagnosis and tailored antimicrobial strategies to improve clinical outcomes [12]. It is capable of causing both healthcare-associated and community-acquired infections. This bacterium possesses multiple virulence factors and has been linked to infections across various organ systems, with a particular impact on the respiratory tract [13]. It is also known for its ability to establish chronic infections through biofilm formation, further complicating treatment. *S. maltophilia* has been isolated from diverse environments and is associated with significant mortality, especially in immunocompromised individuals. Treating *S. maltophilia* is particularly challenging due to its intrinsic resistance to a broad spectrum of antibiotics, with some strains displaying resistance to nearly all available antimicrobial agents, further complicating effective therapy [14]. This highlights the urgent need for novel treatment strategies and alternative therapeutic approaches.

The widespread and often irrational use of antibiotics has contributed to the emergence of bacterial strains resistant to nearly all available treatments. This growing resistance not only increases medical costs and prolongs hospital stays but also poses a serious threat to patient safety, causing anxiety and frustration among policymakers, healthcare providers, and individuals [8,15]. Reducing unnecessary combination therapy, adjusting dosages appropriately, and closely monitoring patients, alongside optimizing antibiotic use, may improve clinical outcomes, minimize side effects, and help curb the emergence of multidrug-resistant bacteria [16]. Since many drugs developed through traditional methods have lost effectiveness due to resistance, innovative approaches to drug discovery are essential. One promising approach involves designing new inhibitors based on bacterial targets, known as pathogen-targeted antibiotics. These antibiotics specifically target bacteria while sparing human cells, enabling timely treatment of infections, lowering healthcare costs, improving patient recovery, and supporting antimicrobial stewardship efforts [17].

**Figure 1:**
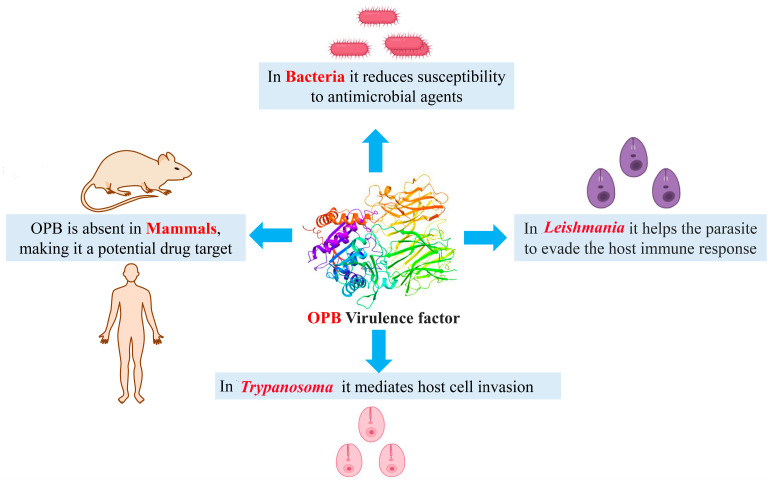
The multifaceted role of OPB as a virulence factor in various organisms.

Aligned with the concept of pathogen-targeted antibiotics, we have identified oligopeptidase B (OPB) as a potential drug target in the Gram-negative bacteria *Serratia marcescens* and *Stenotrophomonas maltophilia*, as well as, for the first time, in the Gram-positive bacterium *Rhodococcus erythropolis*. Found in bacteria and certain parasites but absent in mammals, OPB presents a promising opportunity for the development of novel inhibitors [18]. This proteolytic enzyme (EC 3.4.21.83) belongs to the prolyl oligopeptidase family of serine peptidases (clan SC, family S9) and has been identified in various Gram-negative bacteria and parasites, including *Leishmania* and *Trypanosoma* species. Also known as protease II or trypsin-like protease, it exhibits trypsin-like substrate specificity. The enzyme consists of two domains, a catalytic domain, and a propeller domain, which are connected by a hinge region. It has a dibasic substrate specificity, hydrolyzing peptide bonds on the C-terminal side of basic amino acids (arginine or lysine) in peptides of up to ~30 residues. Due to its strong preference for low-molecular-mass peptides and short-chain oligopeptides, it is functionally classified as an oligopeptidase [19,20,21,22]. OPB, which has not been detected in any mammalian genome, is considered a key virulence factor and a promising drug target for treating trypanosomatid diseases [23]. OPB plays a crucial role in mediating host cell invasion by parasites. Although the precise function of OPB has not yet been fully determined, the targeted deletion of its encoding gene significantly reduces host cell invasion and disrupts infection establishment in mice [24]. The impact of Gram-negative bacterial OPB on reducing susceptibility to antimicrobial agents has been investigated. Studies have shown that overexpression of this enzyme in *Escherichia coli* strains specifically affects bacterial cells, making them more resistant to various proline-rich antimicrobial peptides. Proline-rich antimicrobial peptides have gained attention due to their potent antimicrobial properties, low cytotoxicity, and the relatively low rates of bacterial resistance they elicit. This resistance is particularly concerning, as these peptides are considered promising alternatives to traditional antibiotics. The findings suggest a potential role of OPB in bacterial pathogenicity and its involvement in antimicrobial resistance mechanisms [25,26,27]. Figure 1 illustrates the role of OPB as a virulence factor across various organisms, including *Trypanosoma*, *Leishmania*, and bacteria.

**Figure 2:**
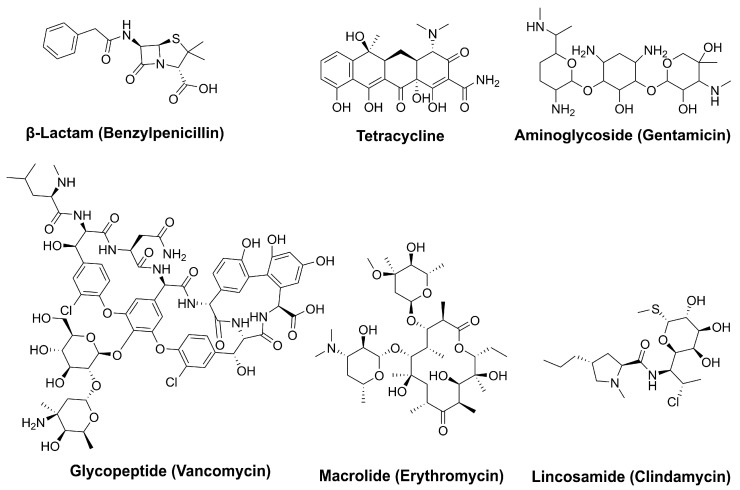
Key microbial natural products with antimicrobial activity.

Natural products have played a crucial role in the development of antimicrobial agents, offering potent and effective treatments against bacterial infections. Due to their diverse sources, ease of extraction, well-established methods of obtaining them, structural and functional biodiversity, and bioactivity against various microbial pathogens and diseases, microbial natural products are considered one of the most crucial elements in drug discovery. In fact, the number of therapeutically active and commercially available compounds derived from microbial sources surpasses those discovered or produced from other sources [28]. Recent studies emphasize that novel natural product antibacterials can be used directly or serve as scaffolds for the design of more effective compounds that bypass early cross-resistance, offering a presumably more sustainable approach for the development of potent antimicrobial agents [29,30]. Figure 2 highlights several significant natural products currently in use.

The discovery of penicillin 100 years ago stands as a prime example, demonstrating how naturally derived compounds can revolutionize medicine. Unlike many synthetic agents, which may cause side effects, natural antibiotics often provide safer and more biocompatible alternatives [31,32]. Despite the emergence of resistance, natural products remain a valuable source for new and effective therapeutics, highlighting their continued significance in combating pathogenic bacteria. Given the dual significance of OPB as a potential drug target and the crucial role of natural products in drug discovery, this study focuses on identifying OPB inhibitors from natural sources with the potential to serve as novel antibacterial agents.

## 2. Results and Discussion

**Figure 3:**
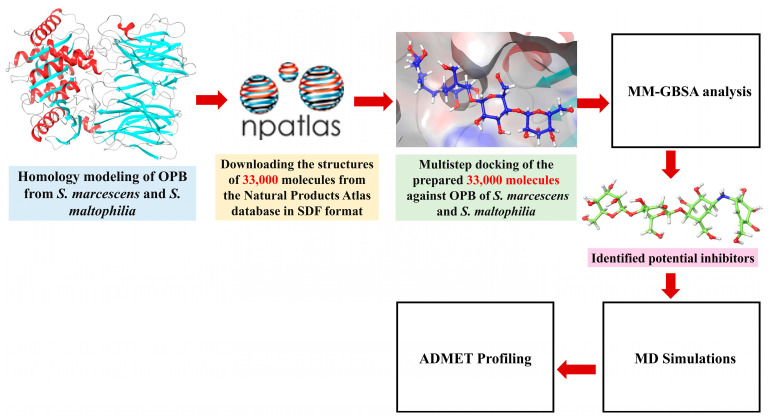
Workflow of virtual screening for OPB inhibitors from the npatlas library.

Antimicrobial resistance, particularly in pathogens such as *S. marcescens* and *S. maltophilia*, represents an escalating global health threat, underscoring the urgent need for new therapeutic strategies. Microbial-derived natural products have emerged as a valuable source of bioactive compounds with the potential to combat resistant bacteria. In this study, we investigated microbial natural products from the Natural Products Atlas (npatlas) library https://www.npatlas.org/ (accessed on 25 February 2025) as potential inhibitors [33]. Our focus was on OPB, a crucial enzyme involved in bacterial virulence, as a molecular for these compounds. Aligned with the strategy of pathogen-targeted antibiotic development, OPB was selected as a potential drug target in the Gram-negative pathogens *S. marcescens* and *S. maltophilia*. As OPB is present in bacteria and certain parasites but absent in mammals, it offers a selective and promising target for novel antimicrobial inhibitors [18]. *S. marcescens* has been increasingly associated with persistent infections due to its ability to form robust biofilms and exhibit resistance to a broad range of antimicrobials, including last-line agents. Importantly, recent studies suggest that certain intracellular proteases, including OPB, may contribute to adaptive responses under antibiotic stress by degrading host defense peptides or modifying intracellular signaling pathways. Investigating OPB in this context provides insight into potential mechanisms of virulence and resistance at the molecular level. Therefore, targeting OPB could represent an innovative anti-virulence strategy that impairs bacterial pathogenic mechanisms without affecting viability, thereby potentially reducing the selection pressure for resistance development [10,11]. For *S. maltophilia*, the rationale stems from its growing classification as a priority pathogen due to its intrinsic resistance to multiple classes of antibiotics, including β-lactams, aminoglycosides, and carbapenems. Unlike *S. marcescens*, *S. maltophilia*’s resistance mechanisms are often driven by efflux pumps and antibiotic-modifying enzymes, yet proteases like OPB remain underexplored in this context. Given the enzyme’s potential involvement in modulating interactions with host peptides and immune components, the study of OPB in *S. maltophilia* could reveal novel therapeutic vulnerabilities. Furthermore, exploring conserved proteolytic targets, such as OPB, across different resistant pathogens enables the identification of broad-spectrum inhibitory scaffolds, a critical goal in next-generation antimicrobial development [12,13]. By selecting these two species, we aimed to establish a model system encompassing clinically significant Gram-negative pathogens that not only pose a therapeutic challenge but may also rely on underexplored resistance-associated enzymes such as OPB. This makes them ideal candidates for structure-based inhibitor design and virtual screening. We believe that targeting problematic bacteria like *S. marcescens* and *S. maltophilia* by inhibiting their OPB holds strong potential in the search for new antimicrobial agents. Given the limited availability of effective treatments for these infections and the fact that these bacteria exhibit multiple virulence factors alongside high levels of antibiotic resistance [10,13,14], the development of alternative therapeutic strategies is critically needed. Since no crystal structures of OPB from these bacteria are available in the Protein Data Bank (PDB), we first performed homology modeling to construct a 3D structure of OPB. Following the screening protocols depicted in Figure 3, we employed various computational methods, including molecular docking, virtual screening, binding free energy calculations, molecular dynamics (MD) simulations, and ADMET profiling. We assessed the binding interactions, stability, and drug-like properties of the compounds. The results highlighted promising candidates with potentially strong antibacterial activity against these resistant pathogens.

### 2.1. Homology Modeling

A BLAST (https://blast.ncbi.nlm.nih.gov/Blast.cgi, accessed on 5 January 2025) search conducted within the experimentally determined Protein Data Bank (PDB) structures revealed an X-ray solved Chain A, OPB from *Serratia proteamaculans* (PDB code 7YWP) as the best template with the highest sequence identity (59.02%), lowest E-value (0.0), a coverage of (95%), and their max and total scores were the same (834). The pairwise alignment of OPB sequences from *S. marcescens* (Query) and *S. proteamaculans* (Subject, Sequence ID: 7YWP), obtained through NCBI BLASTp analysis, is presented in Supplementary Figure S1. The quality assessment of the predicted models and the template structure was carried out using the MolProbity server (https://molprobity.biochem.duke.edu/, accessed on 15 January 2025), which provides insights into stereochemical validity through Ramachandran plot analysis. This analysis categorizes residues into favored, allowed, and disallowed regions, with the proportion of residues in favored regions serving as a strong indicator of structural reliability. The MolProbity evaluation revealed that 96.0% (646/673) of *S. marcescens* OPB model residues were located in favored (98%) regions, while 99.4% (669/673) were within allowed (>99.8%) regions, demonstrating the overall structural soundness of the models. Four outliers were identified: Gln31 (73.0, 105.3), Arg61 (91.1, 76.3), Pro138 (−104.0, 134.9), and Thr335 (72.0, 124.1), with values indicating phi (φ) and psi (ψ) angles. These results closely resemble the template (PDB ID: 7YWP), where 96.1% (648/674) of residues were located in favored (98%) regions, 99.6% (671/674) in allowed (>99.8%) regions, with three outliers: Thr3 (73.0, 105.3), Arg33 (91.1, 76.3), and Ser149 (114.9, 5.6). For the *S. maltophilia* OPB model, 94.4% (637/675) of residues were located in favored (98%) regions, and 98.4% (664/675) were in allowed (>99.8%) regions, with 11 identified outliers. The φ and ψ distributions in the Ramachandran map, generated by the MolProbity server for the OPB targets and the template, are shown in Supplementary Figure S2. The results from Verify3D (https://www.doe-mbi.ucla.edu/verify3d/, accessed on 16 January 2025) provided additional support for the structural accuracy and stability of the OPB models, complementing the outcomes from other validation methods employed in this study. Verify3D evaluates the compatibility of a 3D model with its amino acid sequence by generating 3D–1D profile scores. A model is generally considered reliable if ≥80% of its residues score ≥0.1. In this analysis, both OPB models successfully passed the validation threshold and demonstrated scores comparable to the template structure. The template model (PDB ID: 7YWP) achieved a Verify3D score of 96.15%, while the *S. marcescens* OPB model scored 94.07%. Similarly, the *S. maltophilia* OPB model achieved a score of 94.53%, further supporting the overall quality and reliability of the predicted structures. Supplementary Figure S3 illustrates the Verify3D profile results for the two OPB models and the template structure. The figure visually confirms that the majority of residues in each model achieved acceptable scores (≥0.1), consistent with high structural reliability.

**Figure 4:**
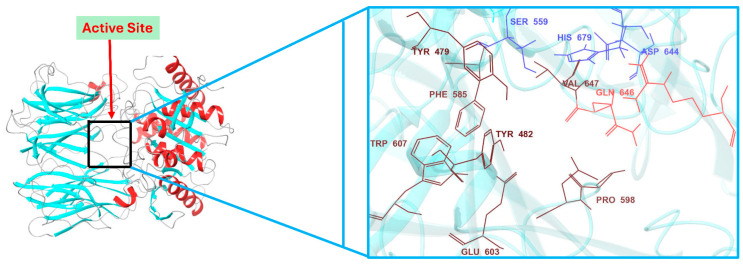
Active site architecture of *S. marcescens* OPB: catalytic triad, hinge region, and key substrate-interacting residues.

The 3D structures of both OPB models adopt a fold similar to that of *S. proteamaculans* OPB, featuring two distinct domains: a β-propeller domain and an α/β-hydrolase catalytic domain. These domains are linked by a flexible hinge region composed of two linear peptide strands, which maintain their structural connection while allowing an open channel for oligopeptides (fewer than 30 residues) to access the active site for processing [20]. The mechanism by which OPBs, also known as serine proteases, cleave various small oligopeptides is facilitated by their catalytic triad (Ser, His, Asp) within the α/β-hydrolase catalytic domain. This domain is covalently linked to a β-propeller domain, with both regions stabilized by a hinge. Figure 4 illustrates the active site of *S. marcescens* OPB, highlighting the catalytic triad, hinge region, and key residues involved in substrate processing. The closed conformation, in which the β-propeller and α/β-hydrolase catalytic domains, anchored by critical residues such as those of the catalytic triad and interdomain salt bridge, are positioned in close proximity, is essential for enzymatic activity. Mutations in these key residues, especially those forming the interdomain salt bridge, can disrupt this conformation and compromise function [34]. Therefore, the integrity of these residues and the closed-state conformation were confirmed in the structural models prior to proceeding with molecular docking and other downstream analyses.

This structural and functional conservation is consistently observed across all identified OPBs in *Leishmania*, *Trypanosoma*, and various bacterial species [20,22,35]. Given the high degree of similarity among these enzymes, inhibitors targeting bacterial OPBs could plausibly be evaluated against OPBs from other organisms to determine their cross-species efficacy. This strategy may facilitate the discovery of broad-spectrum inhibitors with potential therapeutic relevance across diverse pathogenic species. While the OPB sequence from the parasite *Leishmania (Viannia) braziliensis* was previously modeled and used to identify potential synthetic arylated chalcones as anti-parasitic compounds [36], future studies could explore whether the activity of these compounds extends beyond parasites and includes antibacterial effects as well. The current study, however, focuses on identifying OPB inhibitors from natural sources due to several advantages, such as their structural diversity, ease of extraction, and the availability of well-established isolation methods. To evaluate the cross-species inhibitory potential of previously reported OPB inhibitors, we performed molecular docking and MM–GBSA binding free energy calculations for compound C-132, which was identified by Monteiro et al. as a high-affinity inhibitor of *Leishmania* OPB [36]. When docked into the OPB active site of *S. marcescens*, compound C-132 exhibited a relatively low docking score of −5.26 kcal/mol and an MM–GBSA binding energy of −26.76 kcal/mol. These values indicated a significantly reduced binding affinity compared to our top-ranked hits, which demonstrated approximately twice the binding strength based on MM–GBSA calculations. This comparative analysis highlighted the species-specific structural differences in OPB and underscored the superior binding performance of our identified compounds against the *S. marcescens* OPB target. Further examination of the binding interactions of chalcone C-132 within the active site of *S. marcescens* OPB (Supplementary Figure S4) revealed that it occupied the same catalytic pocket as our identified hits and formed a hydrogen bond with the critical catalytic residue Ser559. However, the overall number of stabilizing interactions formed by C-132 was noticeably lower relative to our compounds. Notably, C-132 failed to establish key interactions such as salt bridges and π-cation contacts, which were observed in our top-ranked hits and were believed to have significantly contributed to their enhanced binding affinity and stability within the active site.

### 2.2. Molecular Docking and Binding Free Energy Calculations

**Figure 5:**
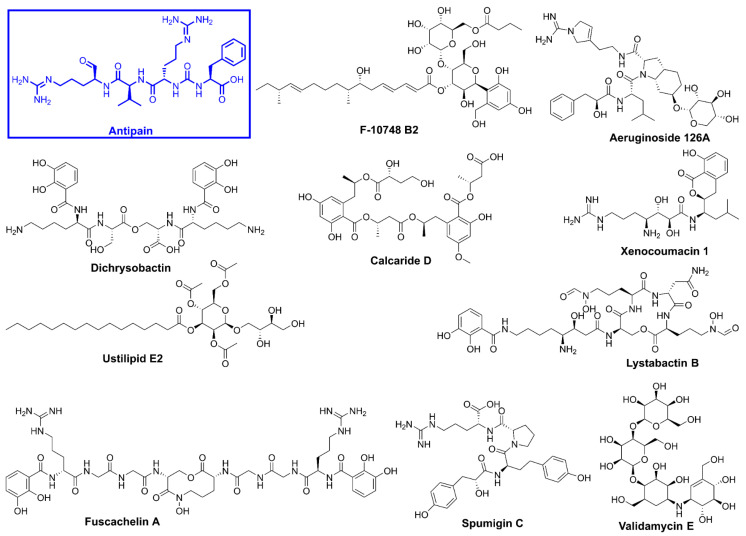
Chemical structures of the top identified hits and the protease inhibitor, antipain.

Molecular docking plays a crucial role in structural molecular biology and computational drug discovery. Its primary objective is to anticipate the most favorable binding interaction(s) between a ligand and a protein with a well-defined three-dimensional structure, aiding in the design of new therapeutics [37]. A comprehensive multistep molecular docking study was performed using the Glide program as a filtering approach to investigate the binding interactions between the screened library and the target protein. The modeled structure of OPB from *S. marcescens* served as the basis for structure-based virtual screening. A microbial-derived compound library containing approximately 33,000 molecules was docked into the catalytic site of OPB. These compounds are expected to function as competitive inhibitors, preventing the binding of natural peptide substrates to the enzyme’s catalytic site and subsequently inhibiting its hydrolysis. The screening workflow began with High Throughput Virtual Screening (HTVS) to identify potential candidates, selecting the top 10% for further evaluation using Glide standard precision (SP) docking. The highest-ranking 10% from this step were then subjected to Glide extra precision (XP) docking, which offers greater accuracy than other docking modes. This systematic filtering approach ultimately yielded 10 promising hits, as detailed in Table 1 and Figure 5, with Glide XP scores ranging from −10.41 to −12.90 kcal/mol. For comparison, the known protease inhibitor, the oligopeptide antipain, used as a reference, exhibited a docking score of −11.42 kcal/mol. This inhibitor, originally isolated from *Actinomycetes*, is widely used in cell biology and biochemistry research laboratories to inhibit various proteases, including OPB, trypsin, papain, plasmin, and other proteases [38]. Taking the docking score of antipain (−11.42 kcal/mol) as a baseline, fuscachelin A (−12.90 kcal/mol) exhibited the highest docking score, indicating the strongest predicted binding to the catalytic site. Validamycin E (−11.89 kcal/mol) and xenocoumacin 1 (−11.71 kcal/mol) also displayed favorable docking scores, suggesting promising interactions that may rival or exceed the reference inhibitor. Aeruginoside 126A (−11.41 kcal/mol) and dichrysobactin (−11.38 kcal/mol) had docking scores close to that of antipain, indicating moderate binding affinity. In contrast, F-10748 B2 (−10.98 kcal/mol), calcaride D (−10.91 kcal/mol), lystabactin B (−10.86 kcal/mol), spumigin C (−10.74 kcal/mol), and ustilipid E2 (−10.41 kcal/mol) showed slightly lower docking scores. Since docking alone may not always provide the most accurate predictions of binding affinity [39], we performed Molecular Mechanics Generalized Born Surface Area (MM–GBSA) calculations to further refine the binding affinity estimates. MM–GBSA is known to offer more accurate results by considering both the binding energy and solvation effects, thereby providing a more reliable assessment of potential inhibitors [40]. The MM–GBSA results (Table 1) revealed that lystabactin B exhibited the most favorable binding, with a value of −81.66 kcal/mol, followed by aeruginoside 126A (−74.17 kcal/mol) and dichrysobactin (−71.40 kcal/mol). Fuscachelin A and validamycin E also showed promising binding energies of −71.52 kcal/mol and −72.52 kcal/mol, respectively. Other compounds, such as xenocoumacin 1 (−68.26 kcal/mol), calcaride D (−67.02 kcal/mol), and ustilipid E2 (−66.60 kcal/mol), also demonstrated significant binding affinities, indicating their potential as inhibitors. Spumigin C and F-10748 B2 showed moderate binding energies of −65.08 kcal/mol and −65.07 kcal/mol, respectively. On the other hand, antipain, the reference inhibitor, had a binding energy of −60.90 kcal/mol, indicating that some of the top hits from this study exhibited more favorable binding affinities compared to the standard inhibitor. These findings suggest that several compounds, including lystabactin B, aeruginoside 126A, and dichrysobactin, could serve as promising lead candidates for further development as OPB inhibitors targeting *S. marcescens*.

**Figure 6:**
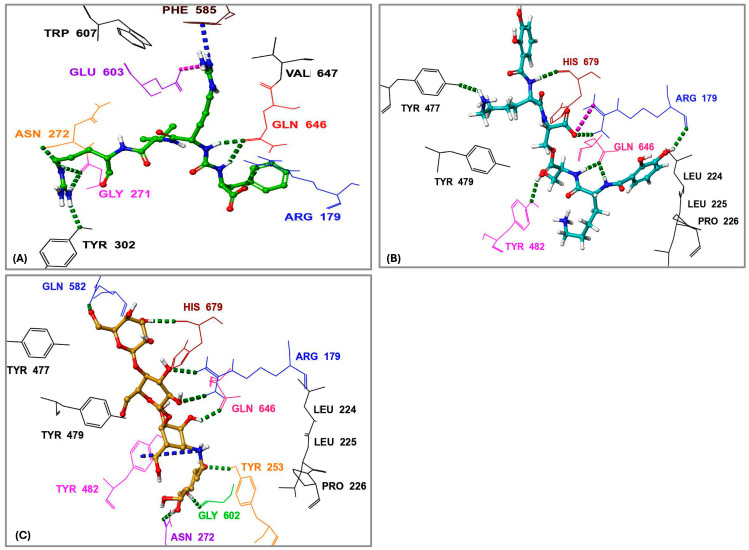
Three-dimensional interactions of the protease inhibitor antipain, dichrysobactin, and validamycin E within the binding site of oligopeptidase B (OPB) from *Serratia marcescens*. (**A**) Antipain; (**B**) Dichrysobactin; (**C**) Validamycin E. Amino acid residues are labeled using their three-letter codes. Hydrogen bonds are represented by green dashed lines, π-cation interactions by dark blue dashed lines, and salt bridges by pink dashed lines. Selected residues involved in hydrophobic interactions are shown in black.

The interactions of the compounds with *S. marcescens* OPB were evaluated based on key binding sites and residues involved (Table 1). Antipain, the reference inhibitor, established multiple hydrogen bonds with key residues. Notably, it formed two hydrogen bonds with Gln646, a residue that typically interacts with Arg179 to create an interdomain salt bridge essential for maintaining the enzyme in its closed, active conformation. It is worth noting that Gln646 corresponds to Gln619 in *S. proteamaculans* OPB, a critical residue required for forming a salt bridge with Arg151. This interaction plays a vital role in stabilizing the catalytic triad, as Gln619 is positioned within the same loop as the catalytic residue Asp617 [41]. Antipain also established hydrophobic interactions with conserved residues among bacterial and protozoan OPBs, including Tyr479, Tyr482, Ala560, Phe585, Pro598, Leu599, Trp607, Val647, and others (Figure 6 and Supplementary Figure S5), contributing to a strong and stable binding profile. Based on the corresponding residues in *S. proteamaculans* OPB [20,41], Tyr479 plays a role in determining substrate specificity and catalysis, while Tyr482 contributes to substrate stability within the S2 binding pocket. Ala560, positioned adjacent to the catalytic Ser559, is involved in defining substrate specificity and processing. Phe585 is critical in the S1 binding pocket and is responsible for enzyme substrate specificity at the P1 site. Pro598 stabilizes the S1 binding pocket, and Leu599, located in the S3 binding pocket, plays a role in substrate stabilization and enzyme specificity at the P3 site. Trp607 helps stabilize Glu603, which, in turn, determines enzyme substrate specificity at the P1 site. Moreover, Val647 interacts with the catalytic triad residues, enhancing substrate processing efficiency. Therefore, antipain is tightly positioned within the enzyme’s active site, effectively blocking natural substrates from accessing the catalytic site. Notably, the key interactions observed between antipain and OPB residues in this study were also present in the co-crystal structures of antipain with *Leishmania major* and *Trypanosoma brucei* OPBs, where the inhibitor effectively blocked enzymatic activity [22,35]. This cross-species similarity in binding patterns suggests that compounds mimicking antipain’s interactions may exhibit broad-spectrum inhibitory potential against both protozoan and bacterial OPBs, a hypothesis that warrants further investigation. Based on this, we believe that inhibitors exhibiting a similar binding mechanism to antipain, or those extending their interactions with additional active site residues, would serve as potent OPB inhibitors. F-10748 B2 formed a crucial hydrogen bond with His679, a key residue of the catalytic triad in the active site. Furthermore, it interacted with the highly conserved Tyr479, which plays a significant role in determining substrate specificity while also establishing substantial hydrophobic interactions. This binding pattern closely resembles the antipain’s profile, particularly through interactions with Tyr479, Tyr482, and Leu599, which contribute to either substrate stability or specificity. Similarly, calcaride D established a crucial hydrogen bond with the catalytic His679, accompanied by π–π stacking interactions that contributed to a unique binding mode. This interaction was further stabilized by both a salt bridge and a hydrogen bond with Gln646, a key residue responsible for the enzyme’s interdomain salt bridge, which is essential for its activity. This was confirmed using a single-residue mutation approach, where altering either of the residues responsible for the enzyme’s interdomain salt bridge, Arg179 or Gln646, disrupted the closed conformation necessary for stabilizing the catalytic triad, thereby compromising enzymatic activity [34]. Moreover, calcaride D engaged in hydrophobic interactions with Tyr479, Tyr482, Val647, Leu599, and Ala560, all of which are crucial for enzyme activity, as previously described.

In a manner similar to calcaride D, the identified inhibitors aeruginoside 126A, dichrysobactin, and validamycin E formed a crucial hydrogen bond with both the catalytic His679 and the residues responsible for the enzyme’s salt bridge, Gln646 and Arg179, which are essential for enzyme stability and activity. In contrast, fuscachelin A also established a crucial hydrogen bond with His679 but did not interact with the residues involved in the enzyme’s salt bridge. However, inhibitors such as spumigin C, ustilipid E2, lystabactin B, and xenocoumacin 1 specifically formed hydrogen bonds with residues responsible for the enzyme salt bridge. Despite these variations, all identified inhibitors behaved similarly to antipain by engaging in hydrophobic interactions with key residues that were also involved in antipain binding. These interactions, first observed between antipain and key OPB residues and later mirrored by the identified inhibitors, are critical for enzyme inhibition. Notably, such interactions have been consistently observed not only in bacterial OPBs but also in OPBs from *Leishmania* and *Trypanosoma*, further reinforcing their conserved role in enzymatic function and inhibition [20,22,35,41]. This highlights the potential to derivatize these inhibitors for broad-spectrum activity across bacterial and protozoan pathogens.

Among all compounds, F-10748 B2, calcaride D, aeruginoside 126A, dichrysobactin, validamycin E, and fuscachelin A, displayed superior interactions (Supplementary Figure S5) with the catalytic residues, indicating their higher potential as OPB inhibitors targeting *S. marcescens*. The 3D interactions of dichrysobactin and validamycin E, as representative examples, along with the protease inhibitor antipain, are provided in Figure 6.

**Figure 7:**
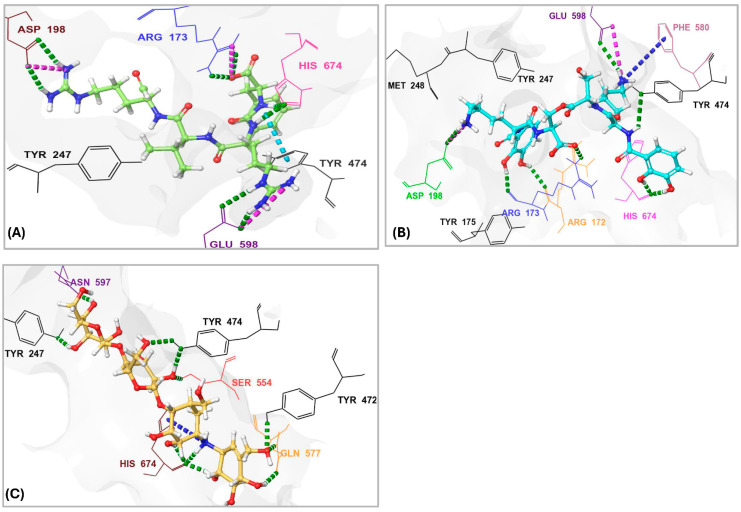
Three-dymensional interactions of the protease inhibitor antipain, dichrysobactin, and validamycin E with the binding site of OPB from *Stenotrophomonas maltophilia*. (**A**) Antipain; (**B**) Dichrysobactin; (**C**) Validamycin E. Amino acid residues are labeled using three-letter codes. Hydrogen bonds are shown as green dotted lines, π-cation interactions as dark-blue dotted lines, salt bridges as pink dotted lines, and some of the interacting residues involved in hydrophobic interactions are colored black.

*S. maltophilia* is an opportunistic pathogen known for its intrinsic resistance to multiple antibiotics, making it a significant challenge in clinical settings. Its ability to form biofilms and cause infections in immunocompromised patients underscores the need for novel therapeutic strategies [42]. Therefore, we performed molecular docking of the top 10 hits originally identified against *S. marcescens* into *S. maltophilia* OPB. This approach aimed to evaluate their binding interactions and determine whether these compounds could serve as potential broad-spectrum OPB inhibitors. By comparing their docking scores and molecular interactions, we sought to identify candidates with strong affinities for both bacterial targets. Such dual-targeting inhibitors could offer promising therapeutic options, particularly in combating multidrug-resistant strains. The docking scores (Table 2) revealed notable variations in binding affinities, with antipain, a well-known protease inhibitor, serving as the reference compound at −10.70 kcal/mol. Dichrysobactin exhibited the strongest binding, with a significantly higher docking score of −14.81 kcal/mol, suggesting it could be a more potent inhibitor. Validamycin E also demonstrated superior binding at −13.11 kcal/mol, while F-10748 B2 and xenocoumacin 1 showed slightly stronger interactions than antipain, with docking scores of −12.34 kcal/mol and −12.13 kcal/mol, respectively. fuscachelin A, with a score of −10.29 kcal/mol, was nearly equivalent to antipain. In contrast, aeruginoside 126A (−8.38 kcal/mol), lystabactin B (−7.64 kcal/mol), spumigin C (−7.26 kcal/mol), and ustilipid E2 (−6.17 kcal/mol) exhibited weaker binding. Calcaride D had the lowest docking score (−5.90 kcal/mol), indicating the weakest interaction. These findings highlight dichrysobactin, validamycin E, F-10748 B2, and xenocoumacin 1 as promising candidates with stronger binding affinities than antipain, suggesting their potential effectiveness against *S. maltophilia* OPB. The MM–GBSA binding free energy calculations provide a more accurate and comprehensive assessment of compound binding compared to docking scores, as they consider both molecular interactions and solvation effects. In this study, antipain, a known protease inhibitor, served as the reference compound with a binding free energy of −27.07 kcal/mol (Table 2). F-10748 B2 demonstrated the strongest binding with a significantly higher binding free energy of −76.59 kcal/mol, making it the most promising candidate, far superior to antipain. Dichrysobactin followed with a score of −71.57 kcal/mol, indicating that it also had strong binding potential, making it an effective candidate. Xenocoumacin 1, with a score of −70.31 kcal/mol, and validamycin E, with −61.36 kcal/mol, both showed comparable binding affinities, outperforming antipain in binding strength. Lystabactin B (−66.26 kcal/mol) and fuscachelin A (−47.53 kcal/mol) exhibited moderate binding affinity, with lystabactin B being notably stronger than antipain. On the other hand, aeruginoside 126A (−43.84 kcal/mol), spumigin C (−40.51 kcal/mol), and ustilipid E2 (−45.76 kcal/mol) demonstrated weaker binding affinities but still performed better than antipain. Calcaride D, with the lowest binding free energy score of −30.67 kcal/mol, showed the weakest binding interaction among the compounds tested. These findings clearly demonstrate that the top 10 identified hits evaluated in this study exhibit binding free energies surpassing that of antipain, highlighting their stronger binding potential as inhibitors against *S. maltophilia* OPB. The interactions of the top 10 identified hits were analyzed and compared to antipain, a known protease inhibitor, to understand their binding mechanisms against *S. maltophilia* OPB. Antipain formed multiple hydrogen bonds with key residues, including two with the catalytic His674, as well as interactions with Arg173 and Gln641, residues involved in the enzyme’s stabilizing salt bridge, and with Glu598. Furthermore, it exhibited a salt bridge interaction with Arg173 and Glu598 and hydrophobic interactions with key residues like Ala555, Tyr474, Tyr477, and Pro593. The binding of antipain to the enzyme also involved π–π interactions with Tyr474. When comparing the interactions of the top 10 hits, it was observed that most of them exhibited stronger or more varied interactions than antipain. For example, F-10748 B2 formed H-bonds with the catalytic His674, Arg173, Gln641, Tyr472, and Gly675 and had hydrophobic interactions with several residues, including the key residue Tyr477. Its interactions with the conserved residues Tyr472 and Tyr477, in particular, suggest a strong binding potential. Ustilipid E2 closely resembled F-10748 B2 and exhibited similar interactions with key residues, including the catalytic His674, Arg173, and Gly675, as well as the conserved residues Tyr474 and Tyr477. Calcaride D also showed multiple H-bonds with several residues and a π–π interaction with Tyr477. The hydrophobic interactions of calcaride D with Tyr474, Tyr477, Leu687, and Val578 were similar to those observed in antipain but involved more residues, indicating a potentially stronger binding affinity. Lystabactin B exhibited interactions more similar to calcaride D, particularly with Tyr474 and Tyr477, but extended its binding to include Arg173, a key residue responsible for the enzyme’s interdomain salt bridge. Dichrysobactin exhibited a more complex interaction profile, forming hydrogen bonds with the catalytic His674, Arg173, and Gln641, as well as key residues Tyr474 and Glu598. It also formed a salt bridge with Glu598 and engaged in an extensive hydrophobic interaction network involving key residues such as Tyr474, Tyr477, Ala555, and Pro593. These interactions suggest a robust binding capability, possibly superior to antipain. Aeruginoside 126A showed H-bonds with the key residues Gly675, Arg173, and Glu598, along with π-cation interactions with Arg173, which were not seen with antipain. Its hydrophobic interactions with the conserved residues Tyr474 and Tyr477 further strengthened its binding affinity. Spumigin C exhibited interactions similar to antipain, forming H-bonds with key residues, including the catalytic His674 and Tyr474. It also displayed π–π and hydrophobic interactions with Tyr477, along with additional hydrophobic interactions with Tyr474. Fuscachelin A demonstrated H-bonds with Arg173, Tyr474, and Glu598. Its hydrophobic interactions with residues such as Pro593, Ala555, Tyr474, and Tyr477 further enhanced its binding affinity compared to antipain. Validamycin E exhibited strong binding affinity by forming two hydrogen bonds with the key catalytic residue Ser554 and three with the other catalytic residue, His674, mirroring the antipain’s interaction with His674. Additional hydrophobic interactions with Tyr474, Tyr477, and Ala555 further contributed to its binding strength. Notably, validamycin E also formed a unique π-cation interaction with His674, which was absent in antipain. Finally, in a manner similar to validamycin E, xenocoumacin 1 exhibited strong binding affinity by forming hydrogen bonds with several key residues, including the catalytic Ser554, Gln641, Tyr474, and Glu598, along with a salt bridge interaction involving Glu598. Its hydrophobic interactions were extensive, engaging residues such as Tyr474, Tyr477, Ala555, and Pro593, further supporting its strong binding potential. The roles of the *S. maltophilia* OPB residues mentioned above are well-documented in both bacterial and protozoan OPBs. For instance, Ser554, Asp639, and His674, corresponding to Ser577, Asp662, and His697 in *L. major* OPB and Ser532, Asp617, and His652 in *S. proteamaculans* OPB, form the catalytic triad essential for enzymatic activity and peptide processing. Similarly, *S. maltophilia* residues Arg173 and Gln641 correspond to Arg664 and Glu179 in *L. major* and Arg151 and Glu619 in *S. proteamaculans*, which are involved in forming the interdomain salt bridge. This structural feature is crucial for stabilizing the enzyme’s closed conformation, allowing peptides shorter than 30 residues to access the active site for processing. In the same manner, conserved residues across bacteria and protozoa, Tyr474, Tyr477, Ala555 (located adjacent to the catalytic serine), Pro593, Glu598, and Gly675, play critical roles in shaping the S1–S3 binding pocket and are likely involved in determining substrate specificity. The observed interactions of antipain, as well as those of the identified inhibitors with key residues of *S. maltophilia* OPB, have been reported in previous studies, confirming the importance of these residues for enzymatic activity. Catalytic residues, those involved in the interdomain salt bridge and stabilization of the enzyme’s active closed conformation, as well as those contributing to substrate specificity, have been validated either through site-directed mutagenesis or by analyzing the disruption of the 3D structure upon inhibitor binding. Notably, these findings are supported by co-crystallized ligands such as antipain, tosyl-L-lysine chloromethyl ketone, and spermine [20,21,22,35,41]. In summary, the top 10 hits demonstrated diverse and stronger interactions than antipain, with many exhibiting more extensive hydrogen bonding, salt bridges, and hydrophobic interactions. These differences suggest that the top 10 hits have a stronger and more robust binding affinity against *S. maltophilia* OPB, making them promising candidates for further development as inhibitors. Particularly, dichrysobactin and validamycin E exhibited more favorable interactions against both *S. marcescens* OPB and *S. maltophilia* OPB, showing superior binding profiles compared to antipain. These enhanced interactions were highlighted in Figure 7 for a clear comparison with antipain.

F-10748 B2 is a fungal-derived antifungal antibiotic known to inhibit 1,3-β-glucan synthase, a key enzyme involved in fungal cell wall synthesis, thereby exhibiting potent antifungal activity [43]. Calcaride D, another fungal metabolite, has shown inhibitory effects against certain Gram-positive bacterial strains [44]; although its exact mechanism of action remains unclear, the current study’s findings indicate inhibitory activity against Gram-negative bacterial OPB. This expands its potential therapeutic applications and may facilitate the development of novel antibacterial agents. Dichrysobactin, a siderophore produced by plant-pathogenic bacteria, and lystabactin B, a marine-derived siderophore, are both known to chelate iron, potentially leading to microbial iron starvation and growth inhibition [45,46]. In this study, both compounds were found to inhibit bacterial OPB, suggesting an additional mode of action beyond iron competition. Similarly, fuscachelin A, another peptide-based siderophore [47], also exhibited OPB inhibitory activity. These findings highlight the multifunctional potential of siderophores and support their further investigation as promising leads for antibacterial agents targeting OPB. Validamycin E, an aminoglycoside primarily used in agriculture to combat fungal infections, and xenocoumacin 1, a compound known for its potent antifungal activity against plant pathogens [48,49], both exhibited inhibitory effects against bacterial OPB in this study. This unexpected antibacterial activity suggests new therapeutic potential for these compounds and highlights opportunities for hit-to-lead optimization in antibacterial drug development. Notably, validamycin E is a carbohydrate-rich natural product classified as a pseudooligosaccharide, well recognized for its broad-spectrum antimicrobial activity. Carbohydrate-based compounds are increasingly valued in medicinal chemistry due to their structural diversity and wide range of bioactivities, including antibacterial, antifungal, antiviral, and anticancer properties [50].

Aeruginoside 126A, a cyanobacterial peptide previously reported to inhibit serine proteases without affecting human plasma plasmin, offers a promising scaffold for the development of selective OPB inhibitors that minimize off-target effects on mammalian systems [51]. Its specificity underscores its potential utility in designing safer antimicrobial agents. Similarly, spumigin C, another cyanobacterial metabolite that is both structurally and functionally analogous to aeruginoside, was also found in this study to inhibit bacterial OPB. The comparable bioactivity of these two compounds highlights a shared pharmacophore that may be exploited for future drug development. Lastly, ustilipid E2, previously described as a microbial surfactant, has been noted for certain derivatives that antagonize dopamine and neurotensin receptors [52]. In the present study, ustilipid E2 demonstrated inhibitory activity against bacterial OPB, suggesting a novel bioactivity profile and presenting a compelling case for the repurposing of ustilipid derivatives as antibacterial agents specifically targeting OPB and potentially other serine proteases.

**Figure 8:**
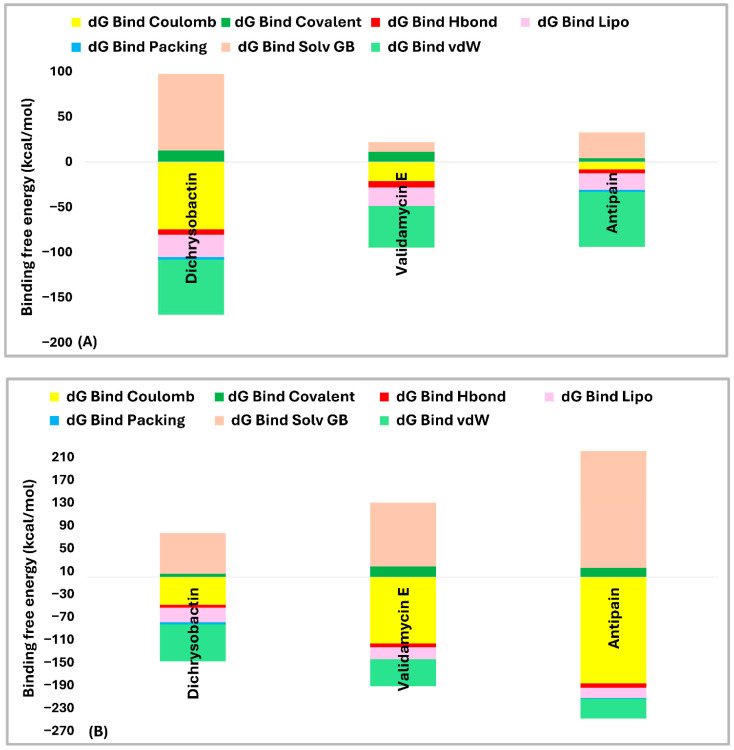
Comparative binding energy breakdown of dichrysobactin, validamycin E, and antipain against OPB from *S. marcescens* (**A**) and *S. maltophilia* (**B**).

Dichrysobactin and validamycin E exhibited stronger and more stable interactions with both targets, suggesting their potential as effective inhibitors of *S. marcescens* and *S. maltophilia* OPB. Due to their promising binding potential, both compounds were selected for an in-depth analysis of their binding free energies to break down the energy terms contributing to their favorable binding to OPB. The analysis of the binding energy contributions for the hits against OPB from *S. marcescens* (Figure 8A) revealed distinct interaction patterns that determined their affinities. Dichrysobactin exhibited a total binding energy of −71 kcal/mol, with van der Waals interactions (−61 kcal/mol), lipophilic contributions (−25 kcal/mol), and hydrogen bonding (−6 kcal/mol) playing key roles in its stabilization. However, a significant solvation penalty (+85 kcal/mol) counteracted these stabilizing effects. Validamycin E demonstrated a slightly stronger binding affinity, with a total binding energy of −73 kcal/mol. Its binding was primarily influenced by van der Waals forces (−46 kcal/mol), lipophilic interactions (−21 kcal/mol), and hydrogen bonding (−7 kcal/mol). While solvation energy (+11 kcal/mol) still presented a destabilizing factor, it was far less significant than in the case of dichrysobactin. Antipain, the reference inhibitor, exhibited the weakest binding among the three, with a total binding energy of −61 kcal/mol. Its binding was driven by van der Waals (−61 kcal/mol), lipophilic (−18 kcal/mol), and hydrogen bonding (−4 kcal/mol) interactions, but it suffered from a considerable solvation energy penalty (+29 kcal/mol), which reduced its overall effectiveness. In *S. marcescens*, validamycin E emerged as the most potent inhibitor, outperforming dichrysobactin, while antipain showed the weakest binding affinity.

In *S. maltophilia* (Figure 8B), the binding energy trends differed significantly. Dichrysobactin remained a strong binder, with a total binding energy of −72 kcal/mol. The van der Waals (−65 kcal/mol), lipophilic (−26 kcal/mol), and hydrogen bonding (−5 kcal/mol) interactions provided stability, while solvation energy (+71 kcal/mol) remained a major destabilizing factor. Validamycin E, however, experienced a notable decrease in its binding affinity, with a total binding energy of −61 kcal/mol, significantly weaker than in *S. marcescens*. Although van der Waals (−48 kcal/mol) and lipophilic (−21 kcal/mol) interactions were still present, a substantial solvation penalty (+112 kcal/mol) severely reduced its binding effectiveness. Antipain exhibited an even greater decline in affinity in *S. maltophilia*, with a total binding energy of −27 kcal/mol. While it retained some favorable van der Waals (−35 kcal/mol) and lipophilic (−18 kcal/mol) contributions, an overwhelming solvation penalty (+205 kcal/mol) drastically weakened its binding, making it the least effective inhibitor against this bacterial OPB.

When comparing the performance of each hit across both OPB enzymes, dichrysobactin demonstrated the most stable binding affinity, with values of −71 kcal/mol in *S. marcescens* and −72 kcal/mol in *S. maltophilia*, confirming its consistency as a potential inhibitor. Validamycin E performed well in *S. marcescens* (−73 kcal/mol) but displayed a significant drop in affinity in *S. maltophilia* (−61 kcal/mol) due to the severe solvation penalty. Antipain, the reference inhibitor, exhibited moderate binding in *S. marcescens* (−61 kcal/mol) but showed a drastic loss of binding affinity in *S. maltophilia* (−27 kcal/mol), highlighting its limited efficacy. In summary, dichrysobactin emerged as the most promising inhibitor across both bacterial species, while validamycin E showed strong potential against *S. marcescens* but struggled in *S. maltophilia*. Antipain, on the other hand, proved to be the least effective inhibitor in both cases, suggesting that it would not be an ideal candidate for OPB inhibition in these bacterial strains. Given that these two compounds demonstrated stronger inhibitory effects than antipain and may possess additional modes of action beyond their known biological roles, they represent promising candidates for further chemical derivatization and functional validation. Their broad-spectrum activity highlights their potential as valuable leads in the development of novel antimicrobial therapies targeting OPBs [45,48,50].

### 2.3. Molecular Dynamics (MD) Simulations

Molecular dynamics (MD) simulations play a crucial role in computational drug discovery by offering detailed insights into the dynamic interactions between biomolecules. Unlike traditional docking studies, MD simulations account for conformational flexibility, solvent effects, and dynamic intermolecular forces over time, offering a more realistic depiction of ligand–target binding [53]. By evaluating stability, binding behavior, and structural fluctuations, MD simulations enhance the validation of potential inhibitors and support the optimization of drug candidates prior to experimental studies. Dichrysobactin and validamycin E exhibited stronger and more stable interactions with both targets, suggesting their potential as effective inhibitors of *S. marcescens* and *S. maltophilia* OPB. Given their promising binding potential, both compounds were selected for MD simulations to assess their stability and interactions. Antipain, a known reference inhibitor, and the apoprotein (Apo) were also included for comparative analysis. Key molecular dynamics (MD) parameters, including root-mean-square deviation (RMSD), root-mean-square fluctuation (RMSF), and protein–ligand contacts, were analyzed throughout the 100 ns simulation run (Table 3 and Table 4).

**Figure 9:**
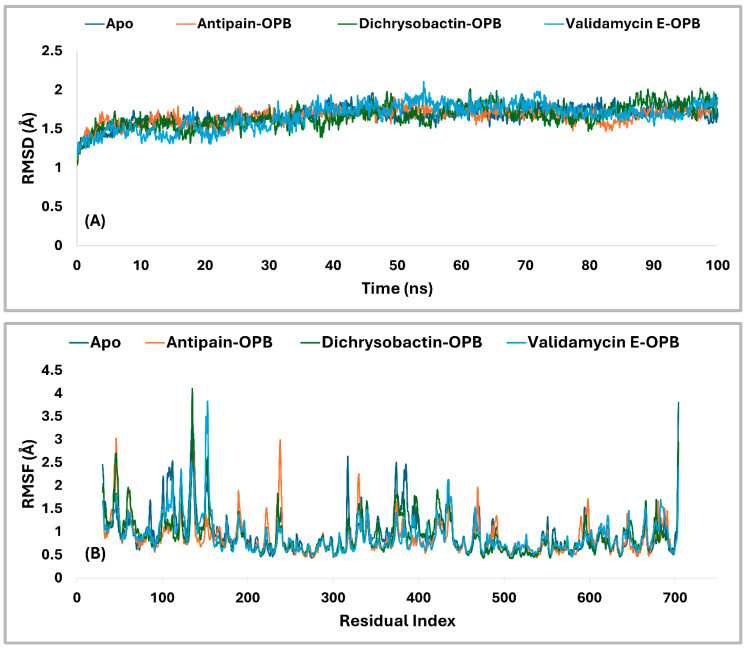
RMSD (**A**) and RMSF (**B**) Profiles (in Å) of the top identified hits (dichrysobactin and validamycin E) in the OPB of *Serratia marcescens*. The analysis was conducted over a 100-ns molecular dynamics simulation, with theApo and reference inhibitor (antipain) included for comparative assessment.

The PL-RMSD analysis (Table 3 and Figure 9A) for *S. marcescens* OPB evaluated the structural stability of the protein in both its Apo (unbound) state and its ligand-bound states (antipain, dichrysobactin, and validamycin E) over the course of the MD simulations. The average RMSD values across all conditions were 1.7 Å, indicating that both the apoprotein and ligand-bound complexes remained within a stable conformational range. The maximum RMSD values slightly varied, with the apoprotein reaching 1.9 Å, antipain and dichrysobactin reaching 2.0 Å, and validamycin E reaching 2.1 Å, suggesting mild fluctuations in structural stability. The minimum RMSD values were similar across all systems, ranging from 1.0 Å (antipain and dichrysobactin) to 1.2 Å (Apo) and 1.1 Å (validamycin E), confirming that none of the ligands caused major destabilization. At the start of the simulation (0–10 ns), all systems exhibited a rapid increase in RMSD, reflecting initial equilibration as the protein–ligand complexes adjusted their binding interactions. The apoprotein (unbound) displayed slightly lower fluctuations compared to ligand-bound systems, indicating that ligand binding introduced minor conformational adjustments. Beyond 10 ns, the RMSD values stabilized within a narrow range, oscillating between 1.5 and 2.0 Å, which confirmed that all ligands remained stably bound within the active site without significant displacement. Dichrysobactin and the apoprotein exhibited the least fluctuation, while antipain and validamycin E showed slightly higher variations. Validamycin E displayed transient peaks exceeding 2.0 Å, aligning with its maximum RMSD of 2.1 Å, indicating greater flexibility in its binding mode. Beyond 50 ns, all systems maintained stable RMSD values, demonstrating that no ligand was unbound from the protein. The Apo structure remained stable throughout, reinforcing the overall rigidity of the OPB protein in both its unbound and bound forms. Dichrysobactin exhibited the highest stability among the bound systems, suggesting a strong and well-retained interaction with the protein. While validamycin E had the highest RMSD fluctuations, its values remained within the acceptable threshold of 2.5 Å, confirming that it did not dissociate from the binding site. The overall RMSD analysis supported the idea that all tested conditions, including the Apo (unbound) protein and ligand-bound states, maintained structural integrity throughout the simulation. The average RMSD of 1.7 Å, with a maximum of 2.1 Å and a minimum of 1.0 Å, indicated that the ligands did not induce significant destabilization. Dichrysobactin and the Apo state showed the greatest stability, while validamycin E displayed the highest flexibility. Despite minor variations, all systems remained within an acceptable RMSD range, validating their stability and potential for further investigation.

The RMSF analysis (Figure 9B) provided insight into the flexibility of residues in *S. marcescens* OPB across different conditions, including the Apo (unbound) state and ligand-bound complexes with antipain, dichrysobactin, and validamycin E. The average RMSF values were relatively low, with the apoprotein exhibiting 1.0 Å and the ligand-bound systems showing slightly reduced fluctuations at 0.9 Å. This indicated that ligand binding had a stabilizing effect on the protein’s overall flexibility, reducing atomic fluctuations across most regions. The maximum RMSF values revealed more pronounced variations in certain regions of the protein. The Apo state reached 3.8 Å, suggesting the presence of highly flexible loops or terminal regions. Antipain-bound OPB exhibited a maximum fluctuation of 3.1 Å, indicating a moderate reduction in flexibility, whereas dichrysobactin showed the highest fluctuation at 4.1 Å, possibly due to interactions that induced localized movements. Validamycin E displayed a maximum RMSF of 3.8 Å, similar to the Apo state, suggesting that its binding did not significantly alter the protein’s flexible regions. The minimum RMSF values were consistent across all conditions, with the Apo, antipain, and dichrysobactin complexes showing a minimum fluctuation of 0.4 Å, while validamycin E exhibited a slightly higher minimum at 0.5 Å. Throughout the simulation, most residues maintained RMSF values below 2.0 Å, signifying that ligand binding did not introduce excessive fluctuations in the protein structure. The apoprotein displayed slightly higher overall flexibility, while the ligand-bound states exhibited localized variations, particularly in loop regions. Dichrysobactin, despite having the highest maximum RMSF, maintained an average fluctuation comparable to the other ligands, suggesting that its effects were localized rather than widespread. Validamycin E, despite minor increases in flexibility, remained within an acceptable fluctuation range. The RMSF analysis confirmed that ligand binding had a stabilizing effect on the protein while allowing for necessary flexibility in dynamic interactions.

Residual fluctuations of the active site residues were also examined to gain insight into the flexibility changes upon ligand binding. The RMSF analysis revealed that the Apo form exhibited moderate fluctuations, with several residues exceeding 1.0 Å, particularly Ser559 (1.079 Å), Ser645 (1.064 Å), Val647 (1.028 Å), and His679 (0.967 Å). Upon binding of antipain, dichrysobactin, and validamycin E, notable changes in RMSF values were observed, reflecting ligand-induced stabilization or flexibility alterations in the active site. Antipain binding generally increased residue fluctuations, with Asp644 and Ser645 showing the highest RMSF values at 1.383 Å and 1.399 Å, respectively, compared to their Apo counterparts. Gln646 also exhibited increased flexibility at 1.028 Å, while other residues like Ser559 (0.668 Å) and Ala560 (0.728 Å) showed reduced fluctuations, indicating stabilization in certain regions. Dichrysobactin binding resulted in variable effects, with Tyr643 experiencing an increase in RMSF to 1.044 Å, whereas other residues like Ser559 (0.66 Å) and Ala560 (0.619 Å) demonstrated decreased flexibility. Validamycin E binding caused the highest fluctuations in Gln646 (1.459 Å), suggesting a more dynamic interaction with this residue, while most other residues maintained fluctuations within a comparable range to the Apo state. Generally, the binding of antipain, dichrysobactin, and validamycin E induced structural adjustments in the active site, influencing the flexibility of key residues. While some regions experienced stabilization, others showed increased motion, particularly in residues Asp644, Ser645, and Gln646, which appeared critical in ligand interactions.

**Figure 10:**
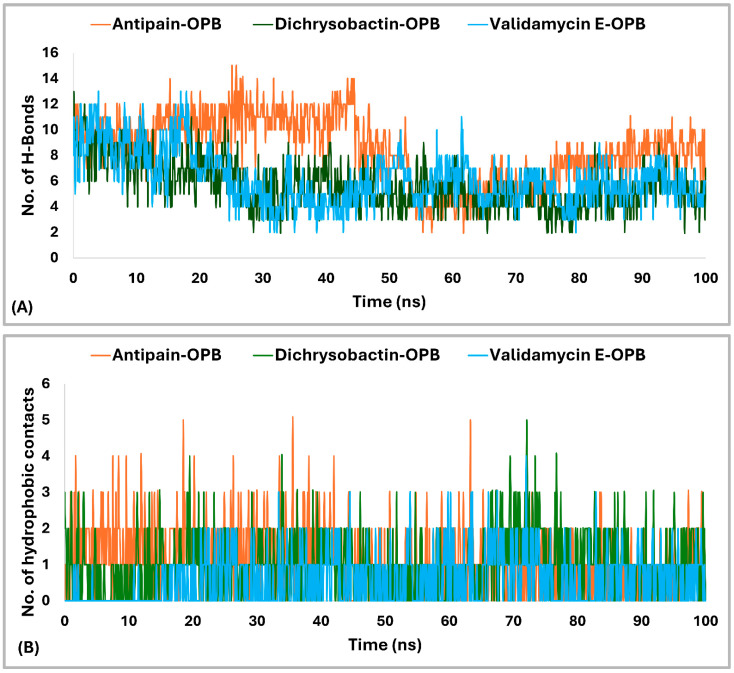
Hydrogen bonding (**A**) and hydrophobic interactions (**B**) of dichrysobactin and validamycin E in comparison with antipain throughout the 100 ns molecular dynamics simulation in the OPB of *S. marcescens*.

The interaction analysis (Figure 10A,B) for *S. marcescens* OPB showed that antipain, the reference inhibitor, formed the highest number of H-bonds with the active site. Specifically, antipain exhibited an average of 8.5 H-bonds, with a maximum of 15 and a minimum of 2.0. In comparison, dichrysobactin formed fewer H-bonds, averaging 5.7 with a maximum of 13 and a minimum of 2.0, while validamycin E showed similar behavior with an average of 6.1, a maximum of 13, and a minimum of 2.0. For hydrophobic interactions, antipain also demonstrated the highest average, with 1.1 interactions and a maximum of 5.0, compared to dichrysobactin (average: 1.0, maximum: 5.0) and validamycin E (average: 0.6, maximum: 4.0). Hydrophobic interactions for all three ligands were minimal, with no ligand exceeding five interactions. Accordingly, the interaction analysis revealed that antipain, as the reference inhibitor, demonstrated the strongest binding interactions with the active site of *S. marcescens* OPB. It formed the highest number of hydrogen bonds and exhibited the most significant hydrophobic interactions compared to dichrysobactin and validamycin E. These findings suggest that antipain’s ability to form more hydrogen bonds likely contributed to its high binding affinity, while its relatively weaker hydrophobic interactions did not significantly hinder its binding effectiveness.

**Figure 11:**
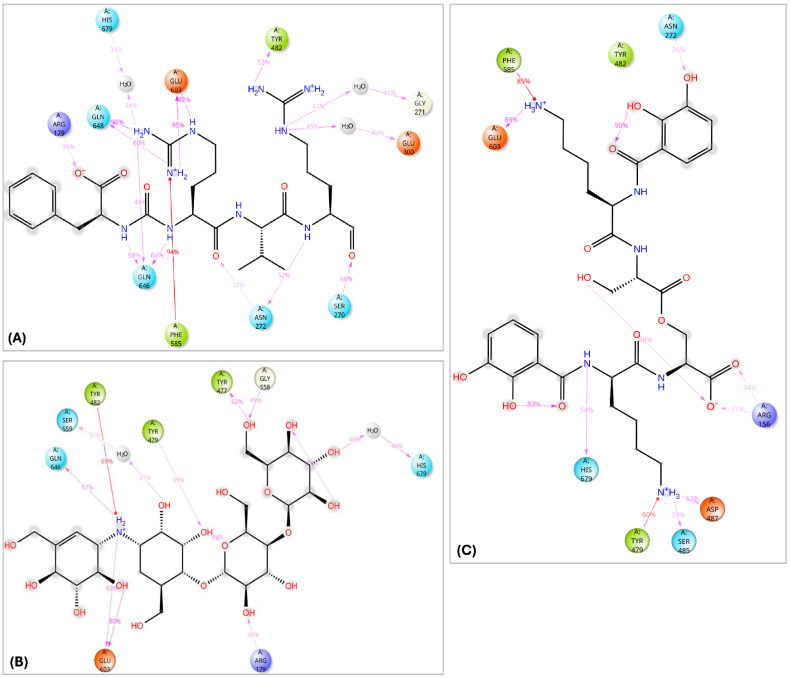
Two-dimensional schematic representation summarizing the key interactions of antipain (**A**), validamycin E (**B**), and dichrysobactin (**C**) with the active site of OPB from *S. marcescens*, observed consistently in over 30% of the MD simulation time.

The interaction analysis for validamycin E, dichrysobactin, and antipain with *S. marcescens* OPB (Figure 11 and Supplementary Figure S6) revealed distinct binding profiles that contributed to their respective binding affinities. For Validamycin, the primary interaction was observed at Ser559 (31%), which suggests a moderate contribution to its binding. A significant interaction was noted at His679, forming a water bridge in 46% of the instances, enhancing the stability of ligand binding. Validamycin also formed strong hydrogen bonds, especially with Arg179 (40%) and Gln646 (57%), indicating critical interactions in the active site. Glu603 showed a high hydrogen bond formation (80%), suggesting its crucial role in Validamycin’s stability. Furthermore, Tyr482 participated in a π-cation interaction (69%), further stabilizing the binding. In the case of dichrysobactin, His679 was again a key player, with interactions observed in 54% of the instances. π-cation interactions were notably high with Phe585 (89%) and Tyr479 (60%), which are significant for the ligand’s stability. Furthermore, Glu603 showed robust hydrogen bonding (89%), indicating its importance in maintaining the structure of the ligand–enzyme complex. These interactions suggest that dichrysobactin’s binding is highly dependent on π-cation interactions and hydrogen bonding, particularly with Glu603 and Phe585. For antipain, the interactions were characterized by strong hydrogen bonds, particularly with Glu603 (95%) and Gln646 (64%), indicating the critical involvement of these residues in binding. Antipain also interacted with Arg179 (39%) and His679 (34%) through hydrogen bonds, providing further stabilization. Moreover, a π-cation interaction with Phe586 (95%) was highly significant, reflecting the importance of this interaction in antipain’s binding. Overall, antipain’s binding profile was distinguished by a combination of strong hydrogen bonds and π-cation interactions, particularly with Glu603 and Phe586, which likely contributed to its higher binding affinity. The comparative analysis of these interactions demonstrates that while all three ligands engaged in hydrogen bonding and π-cation interactions, antipain exhibited the most consistent and strong interactions, particularly at Glu603 and Phe586. Dichrysobactin relied more heavily on π-cation interactions, particularly with Phe585, while Validamycin showed a more balanced interaction profile with notable contributions from Glu603 and His679. These varying interaction patterns suggest different mechanisms for ligand binding and highlight their potential to inhibit *S. marcescens* OPB. The catalytic triad of His679, Ser559, and Asp644 in *S. marcescens* OPB played a crucial role in enzyme catalysis. Ligand interactions, such as those with antipain, dichrysobactin, and validamycin E, significantly influenced the flexibility and stability of this triad. Antipain showed moderate interaction intensity, forming hydrogen bonds with His679 (34%) and Ser559 (39%), stabilizing the active site and potentially affecting catalytic efficiency. Dichrysobactin exhibited stronger interaction intensity, with His679 (54%) and significant π-cation interactions, particularly with Phe585 (89%), stabilizing the enzyme–ligand complex and influencing the catalytic triad’s dynamics. Validamycin also interacted strongly with His679 (46%) and Glu603 (80%), with water bridges and hydrogen bonding, providing a moderate to high level of interaction that likely altered the flexibility of the catalytic triad. In conclusion, the interactions with the catalytic triad residues significantly modulated the stability and flexibility of the active site, with dichrysobactin exhibiting the strongest effect.

**Figure 12:**
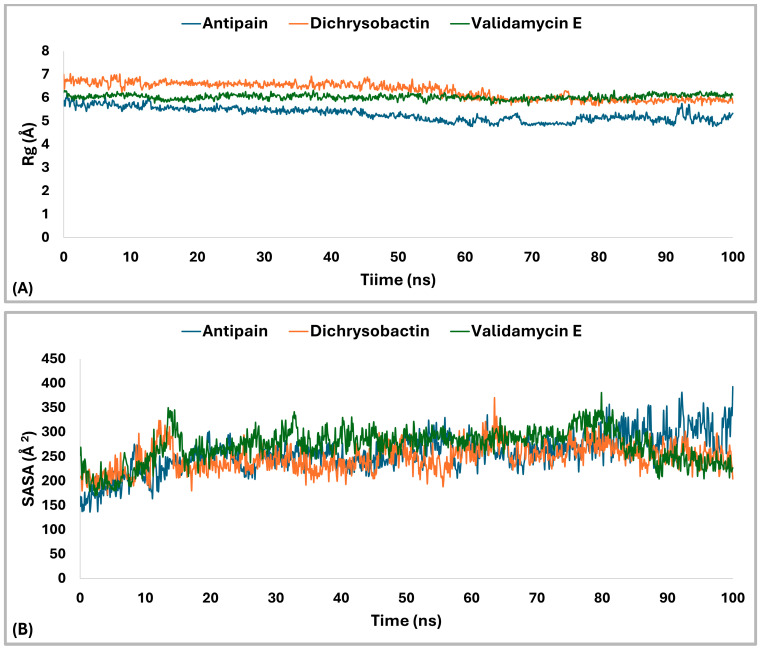
**Rg** and SASA analysis of *S. marcescens* OPB–ligand complexes over 100 ns MD simulation. (**A**) profiles of OPB in complex with antipain (reference, blue), OPB–dichrysobactin (orange), and OPB–validamycin E (green). (**B**) SASA) profiles of OPB in complex with the same ligands.

The radius of gyration (Rg) analysis (Figure 12A) revealed distinct differences in structural compactness among the OPB–ligand complexes. The OPB–antipain complex, used as the reference inhibitor, exhibited the lowest average Rg of 5.29 Å, indicating a highly compact and stable conformation. The OPB–validamycin E complex had an average Rg of 6.03 Å, showing moderate compactness and structural stability. The OPB–dichrysobactin complex displayed the highest Rg average at 6.3 Å, reflecting a more expanded and flexible structure. Throughout the simulation, antipain maintained the most stable Rg profile with minimal fluctuations. Validamycin E showed consistent but slightly higher Rg values, suggesting controlled flexibility. Dichrysobactin’s Rg values fluctuated more widely, indicating dynamic conformational behavior. Compared to the reference inhibitor, both natural compounds induced greater flexibility in the OPB structure. Nonetheless, validamycin E maintained a balance between compactness and flexibility. The increased Rg in the dichrysobactin complex suggested the potential for adaptability but may require optimization for enhanced structural stability.

The Solvent Accessible Surface Area (SASA) analysis (Figure 12B) revealed differences in the surface exposure of OPB when bound to the three ligands. The OPB–antipain complex, serving as the reference, maintained an average SASA of 257.25 Å^2^, indicating a compact and stable conformation. OPB–dichrysobactin showed a slightly lower average SASA of 247.18 Å^2^, suggesting tighter packing around the protein. In contrast, OPB–validamycin E exhibited the highest average SASA at 271.63 Å^2^, implying a more solvent-exposed and flexible conformation. While all three complexes remained generally stable, OPB–validamycin E fluctuated more prominently during the simulation. Dichrysobactin induced modest SASA variation with occasional dips, reflecting transient compacting motions. Antipain, as the reference inhibitor, conferred the most balanced and moderate SASA profile. These differences aligned with the observed radius of gyration trends, further supporting antipain’s stabilizing effect. Validamycin E likely introduced looser protein–ligand interactions, as reflected in its increased SASA. Altogether, antipain demonstrated a consistent reduction in surface exposure compared to the two test compounds.

**Figure 13:**
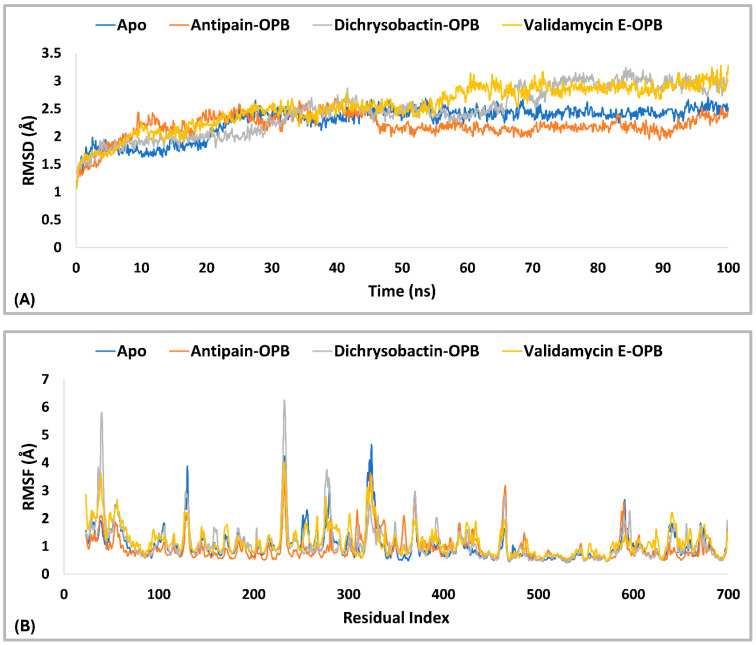
RMSD (**A**) and RMSF (**B**) Profiles (in Å) of the top identified hits (dichrysobactin and validamycin E) in the OPB of *S. maltophilia*. The analysis was conducted over a 100-ns molecular dynamics simulation, with the Apo and reference inhibitor (antipain) included for comparative assessment.

The PL-RMSD analysis (Table 4 and Figure 13A) for *S. maltophilia* OPB assessed the structural stability of the protein in its Apo (unbound) form and when bound to the reference inhibitors antipain, dichrysobactin, and validamycin E during the MD simulations. The PL-RMSD analysis for *S. maltophilia* OPB indicates overall structural stability, with all average RMSD values below 3 Å. The Apo form has an average RMSD of 2.3 Å, while antipain shows the lowest deviation at 2.2 Å. Dichrysobactin and validamycin E have slightly higher average RMSD values (2.5 Å), but they remain within the acceptable range. Maximum RMSD values reach 3.2 Å for dichrysobactin and 3.3 Å for validamycin E, suggesting some flexibility in binding. The minimum RMSD values remain low across all states, ranging between 1.1 Å and 1.3 Å. In the initial phase (0–20 ns), the Apo form showed a sharp increase in RMSD from ~1.2 Å to ~2.2 Å, which indicated the initial relaxation and equilibration of the protein structure. Following this, the RMSD stabilized, suggesting that the Apo form maintained its structural integrity. Antipain–OPB demonstrated a rapid increase in RMSD, reaching ~2.0 Å in the first 10 ns, after which it stabilized with the least fluctuation among all complexes, indicating a stable protein–ligand interaction. Dichrysobactin–OPB showed a steeper increase, reaching ~2.5 Å, which suggested moderate flexibility in the protein–ligand interaction during this phase. Validamycin E–OPB exhibited the highest initial increase, reaching ~2.6 Å, indicating that the complex underwent significant adjustments before stabilization. In the mid-phase (20–60 ns), the Apo form stabilized around ~2.3 Å, exhibiting minimal fluctuations, which confirmed that the unbound protein remained stable without major conformational changes. Antipain–OPB remained the most stable complex, with RMSD values consistently around 2.2 Å, suggesting strong binding and minimal conformational rearrangement. Dichrysobactin–OPB fluctuated between ~2.5–2.8 Å, indicating a more dynamic interaction but still within an acceptable stability range. Validamycin E–OPB showed greater fluctuations, with RMSD occasionally approaching ~3.0 Å, implying that the binding interaction might be less stable or more flexible compared to the other complexes. In the final phase (60–100 ns), the Apo form remained stable, maintaining an RMSD of around 2.3 Å, which indicated no significant conformational shifts. Antipain–OPB demonstrated the greatest stability among all the complexes, maintaining an RMSD of less than 2.5 Å throughout, suggesting that it had the most stable binding interaction with the protein. Dichrysobactin–OPB reached a peak RMSD of ~3.2 Å, but this was still within an acceptable range, indicating that while the interaction remained somewhat flexible, it was still stable. Validamycin E–OPB exhibited the highest fluctuation, with RMSD peaking at ~3.3 Å, suggesting an even more dynamic interaction and possibly weaker binding. In conclusion, all ligand–OPB complexes demonstrated overall structural stability throughout the MD simulations, as indicated by RMSD values that remained below the 3.0 Å threshold, commonly considered acceptable for such analyses. Among them, the antipain–OPB complex exhibited the greatest stability, with the lowest RMSD values and minimal conformational fluctuations, underscoring its strong and consistent binding. Dichrysobactin–OPB also maintained stable dynamics despite displaying moderate flexibility, suggesting a reasonably stable, though slightly less robust, binding interaction. In contrast, validamycin E–OPB showed the highest degree of fluctuation, reflecting a weaker and less stable binding mode. These results suggest that while antipain–OPB forms a highly stable complex, validamycin E–OPB may benefit from structural modifications to enhance its binding affinity and stability.

To further assess the flexibility of the protein–ligand complexes, Protein Root Mean Square Fluctuation (P-RMSF) analysis was conducted, providing insights into the mobility of individual residues and regions within *S. maltophilia* OPB during the 100 ns molecular dynamics simulations. As shown in Table 4 and Figure 13B, the average RMSF values were 1.0 Å for the Apo form, 0.9 Å for antipain–OPB, 1.1 Å for dichrysobactin–OPB, and 1.2 Å for validamycin E–OPB, suggesting generally low flexibility in all complexes. The maximum fluctuations observed were 4.7 Å for the Apo form, 3.6 Å for antipain–OPB, 6.2 Å for dichrysobactin–OPB, and 4.0 Å for validamycin E–OPB, indicating that certain regions of the protein exhibited significant movement, particularly in the dichrysobactin–OPB complex. The minimum RMSF values were 0.4 Å for the Apo form and dichrysobactin–OPB and 0.5 Å for antipain–OPB and validamycin E–OPB, reflecting regions with minimal fluctuation and high stability across all complexes. The Apo state exhibited the highest overall flexibility, while ligand binding generally contributed to structural stabilization. Among the ligands, antipain–OPB demonstrated the strongest stabilizing effect, consistently reducing deviations in critical regions, indicating a robust binding interaction. In contrast, dichrysobactin–OPB induced significant flexibility spikes in residues 36–42 and amplified fluctuations in the 275–280 loop, suggesting localized destabilization. Validamycin E–OPB provided intermediate stabilization, with deviations resembling those of the Apo state, particularly in the C-terminal regions, pointing to weaker binding interactions. The binding site, spanning residues 450–550, remained particularly stable across all conditions, with minimal deviations, confirming its structural preservation regardless of ligand binding. These results emphasize antipain–OPB’s potential as a high-affinity stabilizer while revealing that dichrysobactin–OPB’s unique flexibility pattern may indicate crucial functional or binding regions. Furthermore, the individual flexibility of the most critical residues was analyzed to evaluate their stability in the Apo and ligand-bound states, considering fluctuations below 2 Å as acceptable. Compared to the Apo form, which exhibited an RMSF of 1.329 Å at ARG172, validamycin E–OPB showed the highest fluctuation (1.773 Å), suggesting weaker stabilization, while antipain–OPB significantly reduced it to 0.888 Å. A similar trend was observed for Arg173, where the Apo form had 1.015 Å, but antipain–OPB lowered it to 0.722 Å, whereas dichrysobactin–OPB and validamycin E–OPB increased it to 1.297 Å and 1.469 Å, respectively. Binding site residues Tyr474 and Ser554, which were stable in the Apo form (0.769 Å and 0.741 Å, respectively), exhibited even lower fluctuations in antipain–OPB (0.583 Å and 0.557 Å), confirming its stabilizing effect. Dichrysobactin–OPB and validamycin E–OPB induced slight increases in flexibility at these residues but remained within the acceptable range. The most notable flexibility spikes were observed at Asp639 and Val642, where the Apo form had 1.520 Å and 1.566 Å, respectively, but validamycin E–OPB increased them to 1.749 Å and 2.023 Å, indicating a more dynamic interaction. These results indicate that the antipain–OPB complex exhibited the most significant structural stabilization relative to the unbound (Apo) form of the protein. In contrast, the validamycin E–OPB complex preserved a greater degree of conformational flexibility, especially in regions critical to the protein’s function.

**Figure 14:**
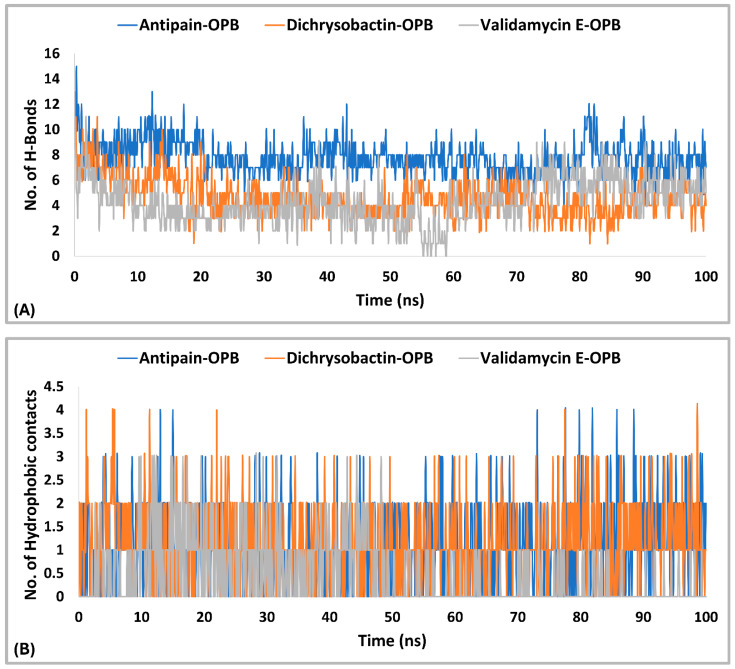
Hydrogen bonding (**A**) and hydrophobic interactions (**B**) of dichrysobactin and validamycin E in comparison with antipain throughout the 100-ns molecular dynamics simulation in the OPB of *S. maltophilia*.

The protein–ligand interactions were analyzed through hydrogen bonding and hydrophobic interactions (Figure 14A,B and Supplementary Figure S7) to assess their contributions to binding stability. Antipain–OPB exhibited the highest average number of hydrogen bonds (7.8), with fluctuations ranging from 4.0 to 15.0, indicating strong and consistent bonding that contributed to its superior stability. Dichrysobactin–OPB had a lower average of 4.6, fluctuating between 1.0 and 11.0, suggesting intermittent interactions. Validamycin E–OPB showed the weakest hydrogen bonding, with an average of 4.3 and occasional losses of hydrogen bonds, reaching 0.0 at certain time points, indicating weak or transient interactions. In terms of hydrophobic interactions, dichrysobactin–OPB had a slightly higher average (1.2) than antipain–OPB (1.1), though both exhibited a similar maximum (4.0), reinforcing their ability to maintain hydrophobic contacts. Validamycin E–OPB displayed the weakest hydrophobic interactions, averaging only 0.5, with a maximum of 3.0, suggesting a predominantly polar binding mode. Overall, the antipain–OPB complex exhibited the most robust and stable interactions, characterized by the highest number of hydrogen bonds and persistent hydrophobic contacts, consistent with its enhanced structural stability. In comparison, dichrysobactin–OPB formed fewer hydrogen bonds but displayed moderately stronger hydrophobic interactions, indicating a more balanced and potentially adaptive binding mode. On the other hand, validamycin E–OPB showed the weakest interaction profile, marked by minimal hydrophobic contacts and highly variable hydrogen bonding, which correlates with its reduced binding stability. These observations suggest that antipain–OPB forms a strong and stable complex, whereas the unstable interaction pattern of validamycin E–OPB points to a need for structural optimization to improve its binding affinity.

**Figure 15:**
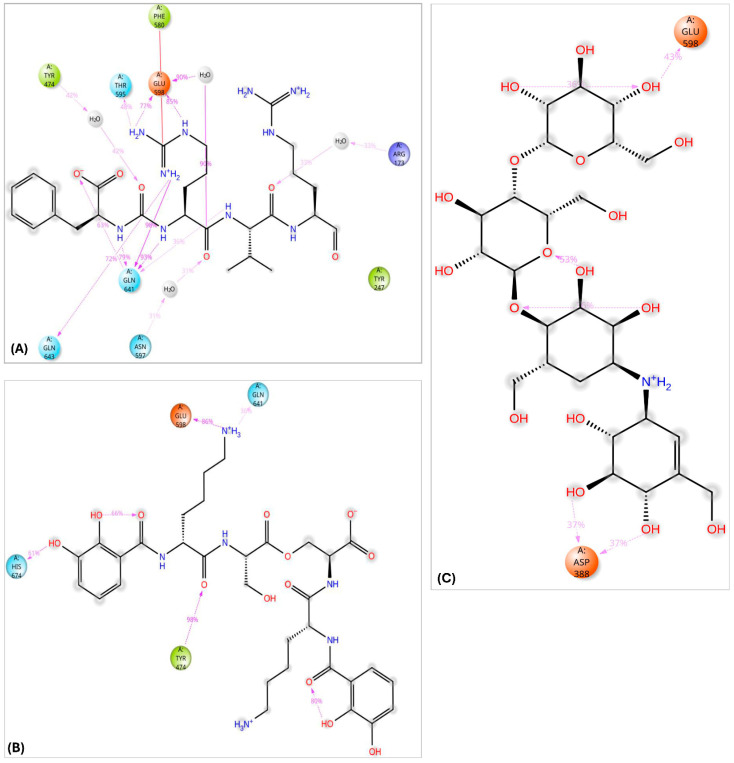
Two-dimensional schematic representation summarizing the key interactions of antipain (**A**), dichrysobactin (**B**), and validamycin E (**C**) with the active site of OPB from *S. maltophilia*, observed consistently in over 30% of the MD simulation time.

The 2D interaction timeline over the 100-ns simulations (Figure 15) provided key insights into the dynamics and stability of ligand–protein interactions for each ligand. In the case of antipain–OPB (Figure 15A), several residues exhibited strong and consistent interactions, particularly with Glu598, which formed a water bridge 90% of the time, demonstrating stable and continuous interaction throughout the simulation. Another critical residue was Gln641, which showed a high interaction frequency of 93%, indicating a stable hydrogen bond that remained largely unperturbed over time. Arg173 interacted with a water bridge 33% of the time, showing less consistency but still contributing to the overall binding stability. Tyr474 and Phe580 were also important in maintaining stability, with Tyr474 interacting 42% of the time, while Phe580 engaged in a π-cation interaction, providing additional stabilization through aromatic interactions. The overall interaction profile of the antipain–OPB complex revealed a well-organized network of hydrogen bonds and aromatic interactions, both of which played a key role in reinforcing its strong and stable binding affinity. For dichrysobactin–OPB (Figure 15B), the interactions were predominantly hydrogen bonds, with Tyr474 forming the most stable interaction (98%), followed by Glu598 (85%) and His674 (61%). These interactions were stable over time, contributing to the overall binding affinity. However, Gln641 exhibited a lower interaction frequency of 36%, indicating a more transient and less consistent interaction. Despite this, the high percentage of hydrogen bonds, especially with Tyr474, underscored the relative stability of the dichrysobactin–OPB complex. Still, the variability in interactions, particularly with Gln641, suggested that dichrysobactin–OPB might experience some fluctuations in its binding mode compared to antipain–OPB. For validamycin E–OPB (Figure 15C), the interactions were weaker and less consistent. Asp388 showed an interaction 37% of the time, and Glu598 interacted 43% of the time, suggesting that these interactions were less stable and more variable than those observed in antipain–OPB and dichrysobactin–OPB. These findings indicate that validamycin E–OPB forms a weaker and more dynamic binding network, likely contributing to its overall lower binding stability. The limited number of persistent interactions suggests that its binding may be less tightly regulated, highlighting the potential need for structural optimization to enhance its affinity and stability. In contrast, antipain–OPB consistently exhibited the most stable and robust interactions throughout the simulation, particularly with key residues such as Glu598 and Gln641, which played crucial roles in maintaining strong hydrogen bonds and aromatic stacking. Dichrysobactin–OPB also demonstrated notable interactions, especially with Tyr474; however, these were slightly more variable, suggesting a less stable binding mode relative to antipain–OPB. Overall, the fluctuating interaction profile of validamycin E–OPB underscores its comparatively unstable binding, emphasizing the importance of improving its interaction network for better performance.

**Figure 16:**
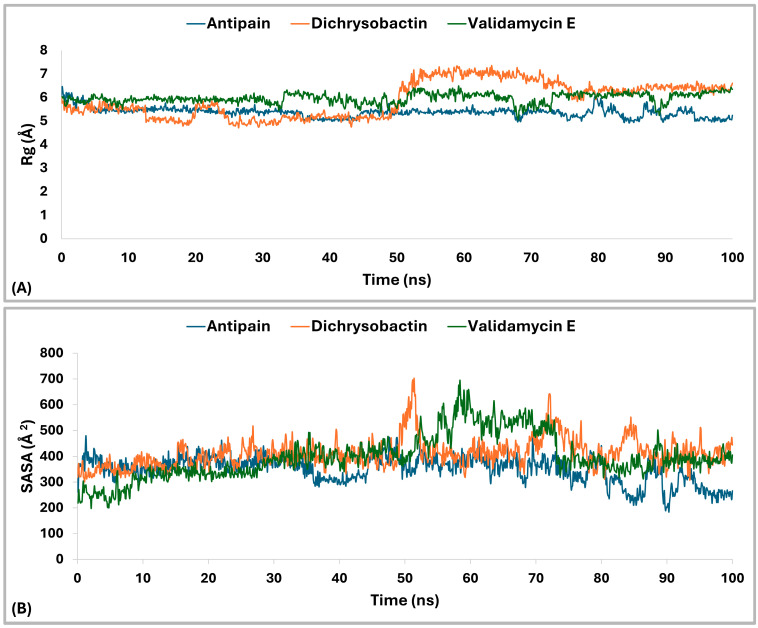
Rg and SASA analysis of *S. maltipholia* OPB–ligand complexes over 100-ns MD simulation. (**A**) Rg profiles of OPB in complex with antipain (reference, blue), OPB–dichrysobactin (orange), and OPB–validamycin E (green). (**B**) SASA profiles of OPB in complex with the same ligands.

The radius of gyration (Rg) plot compared the structural compactness of OPB of S. maltipholia in complex with three ligands: antipain (blue), dichrysobactin (orange), and validamycin E (green) (Figure 16A). Antipain, with the lowest average Rg of 5.36 Å, indicated the most compact and stable complex throughout the simulation. Dichrysobactin showed a moderate average Rg of 5.91 Å, suggesting a less compact structure with slightly more flexibility or fluctuation. Validamycin E exhibited the highest average Rg at 6.3 Å, reflecting the least compact structure, likely due to conformational changes or weaker binding affinity. Around frame 2000, validamycin E showed a marked increase in Rg, indicating structural loosening. Similarly, dichrysobactin experienced a sharp rise in Rg earlier in the simulation, pointing to transient destabilization. Antipain remained mostly stable with only minor fluctuations, reinforcing its role as a reference inhibitor. The order of complex compactness from highest to lowest was antipain > dichrysobactin > validamycin E. This trend is likely correlated with binding strength and conformational stability. Overall, antipain maintained the most rigid conformation of OPB, while validamycin E induced the greatest dynamic changes.

The solvent-accessible surface area (SASA) parameter of the top hits, dichrysobactin and validamycin E, was analyzed with antipain as a reference inhibitor, as it provided insights into protein conformational stability, ligand binding, and solvent interactions. Among the three compounds, antipain exhibited the most stable SASA profile (Figure 16B), maintaining values between 300 and 450 Å^2^ and averaging 349.27 Å^2^ throughout the simulation, which indicated a well-folded, rigid structure with consistent solvent exposure. In contrast, dichrysobactin showed greater variability (300–700 Å^2^), with a peak around 50–60 ns and an average SASA of 411.19 Å^2^, suggesting partial unfolding or transient exposure of hydrophobic regions. Validamycin E demonstrated the least stability, with pronounced fluctuations (200–650 Å^2^), abrupt transitions around 55–60 ns, and an average SASA of 397.53 Å^2^, implying significant structural rearrangements. While antipain’s stable SASA supported its role as a reliable reference inhibitor, the elevated and dynamic profile of dichrysobactin may have reflected functional conformational flexibility. Validamycin E’s erratic SASA behavior raised concerns about its structural integrity and potential for stable binding. The lack of overlapping SASA patterns suggested that each compound induced distinct dynamic effects on the protein. Antipain’s consistent solvent exposure reinforced its suitability as a stable scaffold, whereas the fluctuating behavior of dichrysobactin and validamycin E highlighted the need for further structural optimization. These differences in SASA behavior offered valuable insights into each ligand’s potential efficacy and stability as an inhibitor.

### 2.4. ADMET Profiling

Assessing ADMET (Absorption, Distribution, Metabolism, Excretion, and Toxicity) properties during the early stages of drug discovery is crucial in modern pharmaceutical development. In silico prediction methods offer a cost-effective and time-efficient approach to identifying potential pharmacokinetic and toxicity issues, thereby reducing the risk of failure in later experimental or clinical phases [54]. In this context, we performed an ADMET analysis (Supplementary Table S1) of the top-performing candidate compounds, dichrysobactin, and validamycin E, compared to the reference inhibitor antipain. This evaluation, based on established drug-likeness criteria, revealed several key deviations that could significantly affect the drug development potential of these compounds. Each parameter was systematically assessed against standard benchmarks used in small-molecule drug design, highlighting several structural and physicochemical limitations. While all three compounds remained within acceptable molecular weight ranges, with antipain at 604.7, dichrysobactin at 720.73, and validamycin E at 659.63, concerns emerged regarding their structural flexibility. The number of rotatable bonds for all three (21 in antipain, 28 in dichrysobactin, and 24 in validamycin E) exceeded the recommended threshold of 15, suggesting a high degree of molecular flexibility that may impair target binding affinity and reduce oral bioavailability. Moreover, the presence of reactive functional groups posed potential liabilities, particularly in dichrysobactin and validamycin E, which contained one and two such groups, respectively. These features may compromise chemical stability and increase the risk of off-target effects, thereby limiting their suitability as drug candidates without further optimization. While the number of rotatable bonds is a valuable indicator of molecular flexibility, not all drug-likeness criteria, such as those in Lipinski’s or Veber’s rules, must be strictly met for a compound to be therapeutically viable [55]. Drug-likeness guidelines should be regarded as flexible frameworks rather than absolute exclusionary criteria, as pharmacologically active compounds may exhibit therapeutic potential despite violating one or more conventional parameters. Core physicochemical descriptors, such as hydrogen bond donors and acceptors, molecular weight, lipophilicity (LogP), and the number of rotatable bonds, offer valuable initial insights into oral bioavailability and pharmacokinetic behavior. However, the integration of additional parameters, including stereochemical complexity and three-dimensional conformational flexibility, may provide a more comprehensive and biologically relevant assessment of a compound’s drug-like profile [56].

Polar surface properties and solvation effects further contributed to the unfavorable pharmacokinetic profiles of the identified compounds. The polar surface area (PSA) values of 313.94 Å^2^ for antipain, 357.02 Å^2^ for dichrysobactin, and 299.04 Å^2^ for validamycin E significantly exceeded the 200 Å^2^ limit required for optimal membrane permeability. This was compounded by excessive hydrogen bonding capacity, with donorHB values of 10, 8, and 15, respectively, and acceptorHB values of 12, 15, and 32, all of which dramatically surpassed recommended ranges. These properties largely explained the negligible predicted human oral absorption, reported as 0% for all three compounds, and the extremely low permeability metrics, with predicted apparent Caco-2 cell permeability in quantitative prediction (QPPCaco) and predicted apparent MDCK cell permeability in quantitative predictive models (QPPMDCK) values falling below 0.1 nm/sec. Further analysis of distribution and safety parameters demonstrated a complete inability of these compounds to cross the blood-brain barrier, as indicated by their brain/blood partition coefficients (QPlogBB), which were −6.8 for antipain, −7.86 for dichrysobactin, and −5.58 for validamycin E. Furthermore, the Human Ether-à-go-go-Related Gene (HERG) channel inhibition predictions raised safety concerns, with dichrysobactin (−4.59) and validamycin E (−5.83) classified as high cardiac risk compounds, while antipain (−1.05) showed moderate concern. Serum albumin binding (QPlogKhsa) values also fell outside the ideal range, particularly for validamycin E (−2.69), suggesting potential distribution issues. Metabolic stability and solubility assessments further reinforced the challenges associated with these compounds. The predicted metabolic reactions were excessive, with 9 for antipain, 13 for dichrysobactin, and 15 for validamycin E, highlighting potential metabolic instability. While validamycin E demonstrated favorable aqueous solubility (QPlogS: 0.92), its extreme hydrophilicity (QPlogPo/w: −7.18) rendered it membrane-impermeable. In contrast, antipain and dichrysobactin displayed marginal solubility, with QPlogS values of −4.46 and −3.25, respectively, suggesting that formulation interventions would be necessary to improve their bioavailability. A rule-based assessment confirmed fundamental molecular design flaws, as all three compounds violated multiple drug-likeness criteria. Each molecule exhibited three violations of Lipinski’s Rule of Five and two violations of Jorgensen’s Rule of Three, reinforcing their poor fit within a drug-like chemical space. Moreover, the high “star” counts of 11 for antipain, 11 for dichrysobactin, and 13 for validamycin E indicated that these molecules were extreme outliers compared to conventional drug candidates. Given these findings, specific lead optimization strategies would be necessary to improve the drug-like properties of these molecules. Key priorities for structural modifications include reducing the number of rotatable bonds to enhance rigidity, decreasing the number of polar functional groups to improve permeability, and removing reactive groups to mitigate potential safety risks. Furthermore, the universal poor oral absorption of these compounds suggests that alternative administration routes, such as intravenous or prodrug approaches, should be explored. In the case of validamycin E, structural modifications would be essential to reduce HERG channel affinity and mitigate cardiac toxicity before advancing to preclinical development. This analysis conclusively determined that while antipain, dichrysobactin, and validamycin E may possess interesting biological activity as inhibitors of bacterial OPB, their current physicochemical properties render them unsuitable as drug candidates without extensive structural redesign. Antipain’s relatively better profile, though still inadequate, suggests that its selection as a reference inhibitor was primarily based on its biological activity rather than its drug-likeness. Overall, these data strongly indicate that these molecules require significant medicinal chemistry optimization to transition from chemical tools to viable therapeutic agents. Nonetheless, compounds that violate traditional drug-likeness rules may still hold therapeutic value, particularly when targeting challenging biological systems or using non-oral delivery routes. With appropriate formulation strategies and structural refinements, such molecules can serve as effective leads within the broader, beyond-Rule-of-5 chemical space [57].

## 3. Materials and Methods

### 3.1. Template Selection

A thorough search was conducted to evaluate the availability and quality of OPB structures across structural databases for use as templates in homology modeling. Initial screening revealed three main categories of OPB structures: one derived from a bacterial source and two from parasitic organisms, specifically *Leishmania* major and *Trypanosoma* species. To ensure comprehensive coverage, structural data were collected from the Protein Data Bank (PDB) (https://www.rcsb.org/, accessed on 2 January 2025), and sequence information was cross-referenced with UniProt (https://www.uniprot.org/, accessed on 3 January 2025) and NCBI databases (https://www.ncbi.nlm.nih.gov/, accessed on 3 January 2025). These candidate templates were subjected to preliminary comparative analysis, which included assessment of their functional characteristics, substrate specificity, structural resolution, and overall completeness. The analysis results, including functional relevance and sequence identity, were evaluated with particular attention paid to the conservation of active site residues (Ser, Asp, and His of the catalytic triad) and overall fold similarity, both of which are critical for reliable model building. These insights proved valuable and facilitated the selection of the most suitable template, further supported by BLASTp (https://blast.ncbi.nlm.nih.gov/Blast.cgi, accessed on 5 January 2025) searches against the NCBI non-redundant protein sequences. Subsequently, our previously cloned, sequenced, expressed, purified, and characterized OPBs from *S. marcescens* and *S. maltophilia* were used for template identification [18]. The corresponding protein sequences, available in the NCBI database under accession numbers BAM48923.1 (*S. marcescens*) and BAM48924.1 (*S. maltophilia*), were retrieved in FASTA format. These sequences were aligned against Protein Data Bank (PDB) entries using BLASTp with default parameters (expect threshold = 10, word size = 6, matrix = BLOSUM62), and hits were ranked by sequence identity and E-value.

### 3.2. Sequence Alignment

The combined criteria described in the template selection phase enabled the rational identification of the most appropriate structural template, ensuring both structural compatibility and biological relevance. Sequence alignment was a critical step, as it directly impacts the accuracy and reliability of the resulting homology model. The final template was selected based on a well-aligned sequence with the target, showing high functional conservation with bacterial OPBs. Additional selection criteria included high-resolution crystal structure, sequence identity, lowest E-value, best alignment coverage, and optimal max and total scores. Pairwise sequence alignments were initially performed using BLASTp and Bioedit version 7.2.5, allowing for the rapid identification of top-scoring matches and the evaluation of local and global similarity. The alignments were further analyzed to confirm the conservation of key residues, particularly those forming the catalytic triad. Following initial pairwise alignment and evaluation of database hits, multiple sequence alignment (MSA) was performed to refine the comparison. The MSA included the OPB target sequences, the selected structural template, and a closely related OPB from *Leishmania*, allowing for the assessment of conserved regions and verification of critical residues, particularly those involved in enzymatic activity. This step was essential to ensure that structurally and functionally significant regions, such as the S1 binding pocket and the inter-domain linker, were accurately aligned across all sequences, thereby increasing confidence that the selected template would support the generation of a reliable and functionally accurate 3D model.

### 3.3. Model Building

The OPB target sequences and the selected structural template were imported into the Maestro interface, and homology modeling was performed using Prime, a protein structure prediction module within the Schrödinger suite [58]. The process began with sequence alignment, where Prime aligned the target sequence to the template structure, ensuring the conservation of key functional and catalytic residues. Based on the alignment, an initial model was generated by mapping the target sequence onto the template backbone. Special attention was given to aligning active site residues, including those forming the catalytic triad, to preserve enzymatic functionality in the modeled structure. Loop regions and insertions or deletions not present in the template were modeled de novo using Prime’s loop prediction algorithms. Loop refinement was carried out using knowledge-based sampling and energy scoring to identify the most probable conformations. These regions were subjected to iterative adjustment to ensure backbone continuity and structural compatibility with the surrounding residues. Subsequently, side chains were rebuilt and optimized based on the local structural context to minimize steric clashes and maintain proper hydrogen bonding. Rotamer libraries and environment-specific sampling were used to assign the most favorable side-chain conformations, particularly in the active and substrate-binding regions. Finally, restrained energy minimization was performed using the Optimized Potentials for Liquid Simulations, version 4 (OPLS4) force field to relieve local steric strain and refine bond geometries, especially around loops and active-site residues. Prime applies localized optimization without significantly altering the global fold, improving the overall structural quality. The resulting models were then prepared for further validation and downstream applications, including molecular docking and simulation studies.

### 3.4. Structural Model Validation

The protein backbone torsion angles, phi (φ) and psi (ψ), were validated using the MolProbity server, http://molprobity.biochem.duke.edu/index.php accessed on 15 January 2025, which provides a comprehensive all-atom contact and geometry analysis. This tool evaluates stereochemical quality by generating a Ramachandran plot that classifies residues into favored, allowed, and disallowed regions. The proportion of residues in favored regions serves as a key indicator of model reliability. Furthermore, the OPB models were also validated using the Structure Validation Server SAVESv6.1 (https://saves.mbi.ucla.edu/ accessed on 16 January 2025). Within this server, Verify3D is used, which is a structure evaluation tool that assesses the compatibility of a 3D atomic model with its own amino acid sequence (1D). In this method, each residue is assigned to a structural environment class (e.g., alpha-helix, beta-sheet, loop) and further classified based on its polarity (polar or nonpolar). These classifications are then compared to environment profiles derived from a curated dataset of well-refined, high-resolution protein structures. Each residue’s 3D–1D score reflects how favorable its spatial context is based on the statistical likelihood of observing such an environment for that amino acid. Scores are typically reported in a windowed fashion, identifying segments of the model that may be structurally inconsistent or poorly resolved. Models in which ≥80% of the residues achieve an averaged Verify3D score ≥ 0.1 are generally considered reliable. This two-tiered validation strategy, MolProbity for geometrical and stereochemical integrity and Verify3D for environment compatibility, provides a robust evaluation of the modeled OPB structures. It helps to pinpoint misfolded regions, unusual residue environments, or areas that may require further refinement before downstream applications [59].

### 3.5. Molecular Docking

The homology models of *S. marcescens* and *S. maltipholia* were processed using Schrödinger’s Protein Preparation Wizard [60]. This involved adding hydrogen atoms, assigning proper bond orders, defining zero-order bonds for metals, generating disulfide bridges, and removing water molecules located more than 5 Å from hetero groups. Terminal residues were capped appropriately. Epik was utilized to predict the protonation states of ionizable groups at a physiological pH of 7.0 ± 2.0, ensuring correct hydrogen placement. Hydrogen bond networks and water orientations were optimized, after which the structure was energy-minimized using the OPLS4 force field. The minimization employed the steepest descent algorithm for 100 steps, applying positional restraints on heavy atoms and using an RMSD threshold of 0.3 Å as the stopping condition.

Virtual screening was performed using the Glide module integrated into the Schrödinger software suite 2023-1. A total of 33,000 small molecules from the Natural Products Atlas (npatlas) database were first processed using the LigPrep tool to generate optimized 3D structures and appropriate ionization states [61], with original chirality preserved. Potential ionization states at physiological pH (7.00 ± 2.0) were assigned using Epik, followed by energy minimization via the OPLS4 force field [62].

This database was selected for virtual screening based on several strong justifications. Primarily, it aligns with the study’s goal of identifying natural products of microbial origin that may possess therapeutic potential against infectious diseases [63]. Moreover, it offers extensive coverage of microbial metabolites through well-curated data, making it a robust source for identifying promising bioactive compounds. The platform’s intuitive web interface and regularly updated repository further contributed to its suitability for our research.

A receptor-based grid, centered around the critical residues (Ser559, Asp644, and His679 in the case of *S. marcescens*, and Ser554, Asp639, and His674 for *S. maltophilia* OPB), was generated using default Schrödinger settings to fully enclose the binding pocket and guide docking into the OPB active sites of both species.

Subsequently, these compounds were subjected to the Virtual Screening Workflow (VSW). The Glide (Grid-based Ligand Docking with Energetics) algorithm was employed for molecular docking. This algorithm uses the GLIDEScore scoring function to estimate ligand-binding affinity based on a combination of empirical and force-field-derived terms, including hydrogen bonding, hydrophobic interactions, electrostatic contributions, van der Waals forces, and desolvation penalties. For each ligand, a low-energy conformer was generated. A flexible ligand docking strategy was applied to the prepared compound library using the Virtual Screening Workflow (VSW) protocol, allowing for conformational sampling during the docking process [64]. During docking, ligand conformational flexibility was explicitly considered, allowing for the exploration of multiple rotatable bonds throughout the multistep Glide protocol (HTVS, SP, XP). On the other hand, the receptor structure was maintained as rigid, following conventional docking protocols. Nonetheless, the limited flexibility of residues within the binding site was indirectly addressed during the protein preparation process by optimizing hydrogen-bonding networks and assigning appropriate protonation states. Furthermore, a scaling factor (commonly set to 0.8) was applied to the van der Waals radii of nearby receptor atoms to accommodate slight conformational adjustments and minimize potential steric clashes.

The Virtual Screening Workflow (VSW) employed a tiered docking strategy, progressing through three sequential levels: High-Throughput Virtual Screening (HTVS), Standard Precision (SP), and Extra Precision (XP). At each stage, only the top 10% of ligands based on docking scores were retained for the next phase of refinement. Nonpolar regions were softened by scaling the van der Waals radii to 0.80 with a partial charge cutoff of 0.15. Each ligand yielded one optimal pose, and XP Glide scores were used to prioritize compounds. Molecular interaction analysis in both 2D and 3D formats was performed using the Maestro graphical interface in the Schrödinger suite.

### 3.6. Binding Free Energy Calculations

The top 10 candidates from XP results were further evaluated for binding free energy estimation using the MM–GBSA method via the Prime module [58]. The Pose Viewer Files (PVFs) of these ligands were used as inputs, and the Variable Dielectric Surface Generalized Born VSGB 2.0 solvation model with OPLS4 force field was applied. This method employs a surface-based Generalized Born model that utilizes a Gaussian-shaped surface in place of the traditional van der Waals surface, offering a more refined representation of the solvent-accessible area and enhancing the precision of solvation energy calculations.

Compounds were ranked according to their ∆*G* binding energy scores. The binding free energy of all complexes was calculated using the following equation:

$$∆Gbind=GPL-\left(GP+GL\right)$$

∆*Gbind* is the free energy for binding the ligand (*L*) to the protein (*P*) to form the *PL* complex.

Compounds exhibiting more favorable MM–GBSA binding energy scores than the reference ligand, antipain, were shortlisted for subsequent analysis. Key molecular interactions essential for structural interpretation were further examined and visualized using the tools provided in Schrödinger Maestro.

### 3.7. Molecular Dynamics (MD) Simulations

Molecular dynamics (MD) simulations were carried out on selected docked complexes using the Desmond module (Schrödinger Release 2023-1) to investigate the dynamic behavior, conformational flexibility, and binding stability of protein–ligand interactions over time. Compounds that exhibited binding interactions with key residues in both enzymes of S. marcescens and S. maltophilia, namely, dichrysobactin and validamycin E, along with the reference inhibitor antipain, were selected for simulation, in addition to the Apo forms of OPB from both species. The initial coordinates for the MD simulations were obtained from the previously generated docking poses. Prior to initiating the simulations, each docked complex was loaded into Maestro, the graphical interface used to configure molecular dynamics parameters within the Desmond environment [65]. Each complex was embedded in an orthorhombic water box using the Simple Point Charge (SPC) water model, with a buffer distance of 10 Å from the protein surface in all directions. The system was neutralized by the addition of Na⁺ counterions, and 0.15 M NaCl was added to mimic physiological conditions. The OPLS4 force field was employed to describe all interatomic interactions [62]. Prior to the production runs, each system underwent energy minimization and pre-equilibration using the default Desmond relaxation protocol, which includes restrained minimization and short NVT (constant Number of particles (N), Volume (V), and Temperature (T))/NPT (constant number of particles, pressure, and temperature) equilibration phases. The simulations were run in the NPT ensemble, maintaining a constant temperature of 300 K using the Nosé–Hoover thermostat and a pressure of 1.01325 bar using the Martyna–Tobias–Klein barostat. The Martyna–Tobias–Klein barostat was employed for pressure control with a relaxation time of 2 ps, and the coupling style was set to isotropic to maintain uniform pressure across all dimensions of the simulation box. To ensure reliable molecular dynamics simulations, appropriate cut-off distances were applied. Specifically, non-bonded interactions were truncated at a cut-off distance of 9 Å (the default setting) to balance computational efficiency with accuracy. Each production MD simulation was carried out for 100 ns generating a total of 1000 trajectory frames (recorded every 100 ps. Post-simulation analyses were conducted using the Simulation Interaction Diagram (SID) tool in Desmond. Key dynamic and structural parameters were evaluated, including Root Mean Square Deviation (RMSD), Root Mean Square Fluctuation (RMSF), radius of gyration (Rg), and solvent-accessible surface area (SASA). Additionally, detailed protein–ligand interaction profiles were analyzed to assess the conformational stability, compactness, and surface exposure of the complexes throughout the simulation period.

### 3.8. ADMET Profiling

In this study, the ADMET profiles, along with drug-likeness properties, of dichrysobactin, and validamycin E, along with the reference inhibitor antipain were thoroughly evaluated. The QikProp tool [66], part of the Schrödinger suite, was used to predict a range of descriptors, including molecular weight, logP (octanol/water partition coefficient), logS (aqueous solubility), number of hydrogen bond donors and acceptors, polar surface area (PSA), number of rotatable bonds, and predicted central nervous system (CNS) activity. Furthermore, predictions of human oral absorption and violations of Lipinski’s Rule of Five were considered to estimate drug-likeness. These parameters are critical to assessing the compounds’ potential as orally bioavailable drugs. The compound was considered acceptable if it fell within the recommended range for the majority of descriptors when compared to known drug databases. This profiling supported the compound’s potential as a viable lead for further development.

## 4. Conclusions

This study highlights dichrysobactin and validamycin E as promising natural product inhibitors of oligopeptidase B (OPB), a selective antimicrobial target absent in humans. These compounds showed strong binding affinities, stable interactions in molecular dynamics simulations, and acceptable ADMET profiles. Further experimental validation and lead optimization are warranted to advance their development as potential treatments for antibiotic-resistant bacterial infections.

## Figures and Tables

**Table 1:** Docking scores, binding free energy, and molecular interactions of the top identified hits with the catalytic site residues of *Serratia marcescens* OPB. Data for the protease inhibitor antipain are included for comparison.

Compound	Name	Docking Score (kcal/mol)	MM–GBSA (kcal/mol)	Interactions
Antipain		−11.42	−60.90	H-Bonds:
				Two with Gly271; Asn272; Tyr302; Two with Gln646; Arg179
				Cation-π:
				Phe585 (benzene ring)
				Salt Bridge:
				Glu603^−^ (carboxylate) Charge at pH 7.4
				Hydrophobic:
				Tyr253; Leu599; Pro598; Val647; Tyr302; Ala560; Tyr479; Phe585; Tyr482; Pro226; Leu224; Tyr302; Tyr520; Trp607
NPA004514	F-10748 B2	−10.98	−65.07	H-Bonds:
				Two with Gln582; Glu695; Asn691; Gly680; His679; Ser485; Tyr477; Tyr479
				Hydrophobic:
				Tyr482; Tyr479; Tyr477; Leu475; Met486; Leu599; Val583; Tyr696; Tyr504; Ala251; Tyr253
NPA003411	Calcaride D	−10.91	−67.02	H-Bonds:
				Gln582; Tyr477; Asn691; Arg688; Gly680; His679; Gln646; Ser599.
				Cation-π:
				Arg179⁺ (guanidinium) Charge at pH 7.4
				π–π:
				His679 (imidazole)
				Salt Bridge:
				Arg179⁺; Arg156⁺ (guanidinium) Charge at pH 7.4
				Hydrophobic:
				Tyr477; Tyr479; Tyr482; Met484; Val647; Leu599; Ala560; Met556; Leu689; Val583.
NPA006922	Aeruginoside 126A	−11.41	−74.17	H-Bonds:
				Asn272; Two with Glu300; Two with Arg179; Gln646; Tyr479; His679
				Salt Bridge:
				Glu300^−^ (carboxylate) Charge at pH 7.4
				Hydrophobic:
				Tyr520; Met484; Tyr482; Tyr479; Tyr477; Val583; Leu224; Pro226; Pro598; Leu599; Tyr253; Tyr181
NPA009580	Dichrysobactin	−11.38	−71.40	H-Bonds:
				Arg179; Two with Gln646; Tyr482; His679; Asn691; Tyr477
				Salt Bridge:
				Arg179⁺ (guanidinium) Charge at pH 7.4
				Hydrophobic:
				Leu224; Leu225; Pro226; Tyr181; Met486; Met484; Tyr482; Tyr479; Tyr477; Val583; Ala560; Leu599; Tyr253;
NPA014042	Spumigin C	−10.74	−65.08	H-Bonds:
				Asn272; Arg179; Arg178; Tyr477; Two with Gln646
				Salt Bridge:
				Arg179⁺ (guanidinium) Charge at pH 7.4
				Hydrophobic:
				Tyr253; Leu599; Pro598; Leu224; Pro226; Tyr477; Tyr479; Tyr482; Tyr482; Met484; Met486; Tye181
NPA013544	Ustilipid E2	−10.41	−66.60	H-Bonds:
				Two with Asn272; Tyr479; Tyr253; Gly602; Two with Arg179;
				Hydrophobic:
				Tyr477; Tyr479; Tyr482; Met484; Tyr253; Pro226; Leu599; Val583; Leu694; Ala677
NPA015576	Lystabactin B	−10.86	−81.66	H-Bonds:
				Two with Arg179; Gln646; Ser485; Ser599; Tyr479; Gln582; Thr639; Arg688
				Hydrophobic:
				Tyr477; Tyr479; Ala560 Tyr482; Met484; Met486; Tyr253; Leu224; Leu225; Pro226; Pro598; Leu599; Val583; Tyr181
NPA020353	Fuscachelin A	−12.90	−71.52	H-Bonds:
				Glu603; Asn219; Asp674; Arg688; Asn691; Gly680; His679
				Salt Bridge:
				Asp674^−^ (carboxylate) Charge at pH 7.4
				Hydrophobic:
				Tyr477; Tyr479; Ala677; Tyr482; Met484; Tyr253; Leu224; Pro226; Leu599; Val583
NPA021190	Validamycin E	−11.89	−72.52	H-Bonds:
				Asn272; Gly602; Glu603; Tyr253; Gln646; Two with Arg179; His679; Gln582
				Cation-π:
				Tyr482 (benzene ring)
				Hydrophobic:
				Tyr477; Tyr479; Tyr482; Met484; Tyr253; Leu599; Val583
NPA024799	Xenocoumacin 1	−11.71	−68.26	H-Bonds:
				Gly680; Two with Tyr479; Two with Gln646; Two with Glu603
				Salt Bridge:
				Glu603^−^; Glu651− (carboxylate) Charge at pH 7.4
				Hydrophobic:
				Tyr479; Tyr482; Met484; Met486; Ile597; Met592; Pro598; Leu599; Trp607; Val583; Phe585; Ala560; Val647

**Table 2:** Docking scores, binding free energies, and molecular interactions of the top 10 identified hits against *S. maltophilia* OPB.

Compound	Name	Docking Score (kcal/mol)	MM–GBSA (kcal/mol)	Interactions
Antipain		−10.70	−27.07	H-Bonds:
				Two with Arg173; Two with His674; Two with Glu598; Two with Gln641; Two with Asp198
				π–π:
				Tyr474 (Bezene ring)
				Salt Bridge:
				Arg173⁺ (guanidinium); Glu598− (carboxylate); Asp198− (carboxylate) Charge at pH 7.4
				Hydrophobic:
				Tyr247; Ala555; Met481; Met479; Tyr477; Tyr474; Ile592; Pro593; Leu594; Val642
NPA004514	F-10748 B2	−12.34	−76.59	H-Bonds:
				Two with Tyr472; His674; Gly675; Gln577; Ser480; Gln641; Arg173
				Hydrophobic:
				Tyr472; Tyr477; Met479; Met481; Tyr484; Val578; Ala489; Leu687; Tyr691; Tyr499; Tyr247; Leu594
NPA003411	Calcaride D	−5.90	−30.67	H-Bonds:
				Two with Gln577; Tyr484; Arg683; Tyr472; His264; Asn597
				π–π:
				Tyr477
				Salt Bridge:
				Arg683⁺ (guanidinium) Charge at pH 7.4
				Hydrophobic:
				Leu687; Tyr484; Met481; Met479; Tyr477; Tyr474; Tyr472; Tyr247; Leu594; Val578
NPA006922	Aeruginoside 126A	−8.38	−43.84	H-Bonds:
				Gly675; Two with Arg173; Tyr247; Asn597; Glu598
				Cation-π:
				Arg173⁺ (guanidinium) Charge at pH 7.4
				Hydrophobic:
				Val578; Leu687; Tyr472; Tyr474; Tyr477; Met479; Leu594; Tyr247; Tyr175; Tyr148
NPA009580	Dichrysobactin	−14.81	−71.57	H-Bonds:
				Asp198; Two with Arg173; Arg172; Tyr474; Two with His674; Glu598; Gln641
				Cation-π:
				Phe580 (Benzene ring)
				Salt Bridge:
				Asp198− (carboxylate); Glu598− (carboxylate) Charge at pH 7.4
				Hydrophobic:
				Ile250; Met248; Leu218; Tyr247; Pro593; Leu594; Tyr175; Tyr472; Tyr474; Tyr477; Met479; Val462; Phe580; Val578; Ala555
NPA014042	Spumigin C	−7.26	−40.51	H-Bonds:
				Two with Glu686; Gln577; Tyr472; His674; Tyr474; Asn597
				π–π:
				Tyr477 (Phenol ring)
				Salt Bridge:
				Glu686− (carboxylate) Charge at pH 7.4
				Hydrophobic:
				Tyr472; Tyr474; Tyr477; Met479; Met481; Leu594; Tyr247; Val578
NPA013544	Ustilipid E2	−6.17	−45.76	H-Bonds:
				Gln577; Tyr472; His674; Arg173; Gly675
				Hydrophobic:
				Leu687; Val578; Tyr472; Tyr474; Tyr477; Met479; Met481; Tyr247; Tyr515; Leu594
NPA015576	Lystabactin B	−7.64	−66.26	H-Bonds:
				Glu686; Asn669; Gln577; Arg683; Tyr472; Two with Arg173; Asn597; His264
				Hydrophobic:
				Leu687; Val578; Tyr472; Tyr474; Tyr477; Met479; Met481Tyr247; Leu494
NPA020353	Fuscachelin A	−10.29	−47.53	H-Bonds:
				Two with Arg173; Tyr474; Glu598; Two with Gly676
				π–π:
				Tyr148 (Phenol ring)
				Salt Bridge:
				Glu598− (carboxylate) Charge at pH 7.4
				Hydrophobic:
				Phe113; Tyr119; Pro120; Val139; Val151; Phe149; Tyr148; Ala672; Tyr247; Trp602; Phe580; Leu594; Pro593; Ile592; Ala555; Val642; Tyr175; Tyr474; Tyr477; Met479
NPA021190	Validamycin E	−13.11	−61.36	H-Bonds:
				Two with Gln577; Tyr472; Tyr474; Ser554; Tyr247; Asn597; Three with His674
				Cation-π:
				His674 (Imidazole ring)
				Hydrophobic:
				Leu687; Val578; Tyr472; Tyr474; Tyr477; Met479; Ala555; Tyr247; Leu594; Val578; Leu687
NPA024799	Xenocoumacin 1	−12.13	−70.31	H-Bonds:
				Two with Tyr474; Ser554; Two with Gln641; Two with Glu598
				Salt Bridge:
				Glu598^−^ (carboxylate); Glu646− (carboxylate) Charge at pH 7.4
				Hydrophobic:
				Tyr474; Tyr477; Met479; Met481; Val578; Phe580; Ile592; Pro593; Leu594; Val642; Met587; Trp602; Ala555

**Table 3:** Molecular Dynamics (MD) simulation parameters: RMSD, RMSF, H-Bonds, and hydrophobic interactions of the top-performing hits (dichrysobactin and validamycin E) in comparison with the apoprotein and the reference inhibitor antipain in the OPB of *Serratia marcescens*.

PL-RMSD (Å)				
*S. marcescens* OPB	Apo	Antipain	Dichrysobactin	Validamycin E
average	1.7	1.7	1.7	1.7
maximum	2.0	1.9	2.0	2.1
minimum	1.2	1.0	1.0	1.1
P-RMSF (Å)				
average	1.0	0.9	0.9	0.9
maximum	3.8	3.1	4.1	3.8
minimum	0.4	0.4	0.4	0.5
H-bond				
average	-	8.5	5.7	6.1
maximum	-	15.0	13.0	13.0
minimum	-	2.0	2.0	2.0
Hydrophobic				
average	-	1.1	1.0	0.6
maximum	-	5.0	5.0	4.0
minimum	-	0.0	0.0	0.0

**Table 4:** Molecular Dynamics (MD) simulation parameters: RMSD, RMSF, H-bonds, and hydrophobic interactions of the top-performing hits (dichrysobactin and validamycin E) in comparison with the apoprotein and the reference inhibitor antipain in the OPB of *S. maltophilia*.

PL-RMSD (Å)				
*S*. *maltophilia* OPB	Apo	Antipain	Dichrysobactin	Validamycin E
average	2.3	2.2	2.5	2.5
maximum	2.7	2.7	3.2	3.3
minimum	1.2	1.1	1.3	1.1
P-RMSF (Å)				
average	1.0	0.9	1.1	1.2
maximum	4.7	3.6	6.2	4.0
minimum	0.4	0.5	0.4	0.5
H-bond				
average	-	7.8	4.6	4.3
maximum	-	15.0	11.0	13.0
minimum	-	4.0	1.0	0.0
Hydrophobic				
average	-	1.1	1.2	0.5
maximum	-	4.0	4.0	3.0
minimum	-	0.0	0.0	0.0

## Data Availability

The study’s original contributions are available within this article and its Supplementary Material. For additional information, please contact the corresponding authors.

## Supplementary Materials

The following supporting information can be downloaded at https://www.mdpi.com/article/10.3390/ph18050709/s1, Figure S1: Pairwise alignment of OPB sequences from *S. marcescens* and *S. proteamaculans* via BLASTp analysis; Figure S2: Ramachandran plots of OPB targets and template showing φ and ψ torsional angle distributions; Figure S3: Verify3D structural validation profiles for OPBs; Figure S4: 2D interaction map of compound C-132 within the catalytic pocket of Serratia marcescens oligopeptidase B (OPB), highlighting key binding interactions; Figure S5: 2D interaction map of the top five inhibitors (calcaride D, aeruginoside 126A, dichrysobactin, validamycin E, and fuscachelin A) along with the reference inhibitor antipain, highlighting their binding interactions with the catalytic site of *Serratia marcescens* OPB; Figure S6: Histogram representation of the interactions between antipain (A), dichrysoisobactin (B), and validamycin E (C) with *S. marcescens* OPB throughout the simulation run; Figure S7: Histogram representation of the interactions between antipain (A), dichrysoisobactin (B), and validamycin E (C) with *S. maltophilia* OPB throughout the simulation run; Table S1: Physicochemical and pharmacokinetics descriptors of the identified hits calculated with Qikprop.
