# Supplementary material for: Identification of Bacterial Oligopeptidase B Inhibitors from Microbial Natural Products: Molecular Insights, Docking Studies, MD Simulations, and ADMET Predictions
Source: Pharmaceuticals (Basel). 2025 May 11;18(5):709. doi: 10.3390/ph18050709 (PMC12114661; doi:10.3390/ph18050709)
Supplement: Supplementary file 1 [file pharmaceuticals-18-00709-s001.zip › pharmaceuticals-3617412-supplementary.pdf]

Supplementary Materials

Identification of Bacterial Oligopeptidase B Inhibitors from Microbial Natural Products: Molecular Insights, Docking Studies, MD Simulations, and ADMET Predictions

Malik Suliman Mohamed[1]*, Tilal Elsaman[2]*, Magdi Awadalla Mohamed[2], Eyman Mohamed Eltayib[1], Abualgasim Elgaili Abdalla[3], Mona Timan Idriss[4]

[1]*Department of Pharmaceutics, College of Pharmacy, Jouf University, Sakaka 72388, Saudi Arabia*
[2]*Department of Pharmaceutical Chemistry, College of Pharmacy, Jouf University, Sakaka 72388, Saudi Arabia*
[3]*Department of Clinical Laboratory Sciences, College of Applied Medical Sciences, Jouf University, Sakaka 72388, Saudi Arabia*
[4]*Department of Nursing College, Northern Private College of Nursing, Arar 73312, Saudi Arabia*

*Author to whom correspondence should be addressed:
Malik Suliman Mohamed msmustafa@ju.edu.sa
Tilal Elsaman telbashir@ju.edu.sa

Supplementary Materials

Chain A, Oligopeptidase B [Serratia proteamaculans]

Sequence ID: **7YWP_A** Length: 676 Number of Matches: 1
Range 1: 3 to 673

Score	Expect	Method	Identities	Positives	Gaps	Frame
834 bits(2155)	0.0()	Compositional matrix adjust.	396/671(59%)	492/671(73%)	1/671(0%)	

```
Query  32    PPLAERAPKALTAHGETRTDDYYWLRDDSRKEQKVLNYLKAENRYTEQMMAPYQKLRATL  91
             PP AE+ P  +T HG+TR DDYYWLRDD R + +VL+YL+AEN +T+  + P Q LR TL
Sbjct  3     PPKAEKRPYPITTHGDTRVDDYYWLRDDERTDPQVLDYLQAENAFTDAALKPQQALRETL  62

Query  92    YQEMLGRMSPDDRSVPYQLNGYRYQESYAAGKEFARYQRQALTADAPWQTLLDANQRAAG  151
             Y+EM+ R+   + SVPY  +GYRYQ  +  G E+A Y RQ       W TL+D NQRA
Sbjct  63    YEEMVARIPQQEHSVPYVRHGYRYQTRFEPGNEYAIYVRQPQAESEHWDTLIDGNQRAEQ  122

Query  152   HAYYRLGAMDISRDNRRLAVAEDLQGRRQYRISLRELGSERWSPETLENTSGNMVWANDN  211
               +Y LG +++S DN++LAVAED   RRQY I  + L  + W+ E LENTSG+  WAND+
Sbjct  123   REFYTLGGLEVSPDNQKLAVAEDFLSRRQYDIRFKNLSDDSWTDEVLENTSGSFEWANDS  182

Query  212   QTLFYVRNHPQTLLPYQVYRHQYGTPTAEDKLVYQENDPAFYLSLGRSSSRDYLILTISG  271
              T++YVR H +TLLPYQVYRH  GT    D+L+Y+E D  FY+ L +++S  ++++ +S
Sbjct  183   ATVYYVRKHAKTLLPYQVYRHVVGTDPQLDELIYEEQDDTFYVGLEKTTSDRFILIHLSS  242

Query  272   NTTSEVRLIDANQPQREPQLFAARQNGREYYLDHYRGEFYLRSNHQDPNFGLYHTAAAGK  331
              TTSE+ L+DA++    PQ+F  R+   EY +DHY   FY+RSN    NFGLY +  A +
Sbjct  243   TTTSEILLLDADRADSTPQMFVPRRKDHEYGIDHYHQHFYIRSNKDGKNFGLYQSEQADE  302

Query  332   P-WQTLIAPQAQHEVESFSLFRDWLVVQERANGLVQLRQISWDGKTERAIPFDDASYMAW  390
               WQTLIAP+ +  +E FSLFRDWLVV+ER+ GL QLRQI W     + I FDD +Y  W
Sbjct  303   AQWQTLIAPRIEVMLEGFSLFRDWLVVEERSEGLTQLRQIHWQSGEVKRIAFDDPTYTTW  362

Query  391   LGYNPEPDSDRLRYGYSAMTTPTRTYEWDLNKGERTLLKQQEVKGVDPSLYHSERIWIAA  450
             L YNPEP+++ LRYGYS+MTTPT  YE +L+  ER +LKQQEVK   P  Y SER+W+ A
Sbjct  363   LAYNPEPETELLRYGYSSMTTPTTLYELNLDSDERVMLKQQEVKNFTPENYRSERVWVKA  422

Query  451   RDGVKVPVSLVYRTSLFKNGHNPLLVYGYGAYGMSMDPAFSANRISLLDRGFAYALIHVR  510
             RDGV+VPVSLVYR   F  G NPL+VYGYG+YG SMDPAFSA+R+SLLDRGF + L H+R
Sbjct  423   RDGVEVPVSLVYRHDSFARGTNPLMVYGYGSYGSSMDPAFSASRLSLLDRGFVFVLAHIR  482

Query  511   GGGELGQRWYKQGKLTHKPNSFNDFIDATQALINDGYGQPGRIYAMGGSAGGLLMGAVIN  570
             GGGELGQ WY+ GKL  K N+FNDFID T+ALI  GYG   R++AMGGSAGGLLMGAVIN
Sbjct  483   GGGELGQLWYEDGKLFKKQNTFNDFIDVTEALIAQGYGDAKRVFAMGGSAGGLLMGAVIN  542

Query  571   QAPQLYNAVVAQVPFVDVVTTMLDDSIPLTTGEYEEWGNPHQPAAYALMKSYSPYDNVRR  630
             QAP+L+N +VAQVPFVDVVTTMLD+SIPLTTGEY+EWGNP+Q A Y  +  YSPYD V+
Sbjct  543   QAPELFNGIVAQVPFVDVVTTMLDESIPLTTGEYDEWGNPNQQAYYDYILQYSPYDQVKA  602

Query  631   QRYPNLLVTSGLYDSQVQYWEPAKWVAKLRRFKQGDSLLLLSTDMTAGHGGKSGRLARLE  690
             Q YP++LVT+GL+DSQVQYWEPAKWVAKLR  K  D  LLL TDM +GHGGKSGR    E
Sbjct  603   QDYPHMLVTTGLHDSQVQYWEPAKWVAKLRELKTDDRQLLLYTDMDSGHGGKSGRFKAYE  662

Query  691   NGALEYAFILA  701
             + ALEYAFILA
Sbjct  663   DIALEYAFILA  673
```

Figure S1: Pairwise alignment of OPB sequences from *S. marcescens* and *S. proteamaculans* via BLASTp analysis

Supplementary Materials

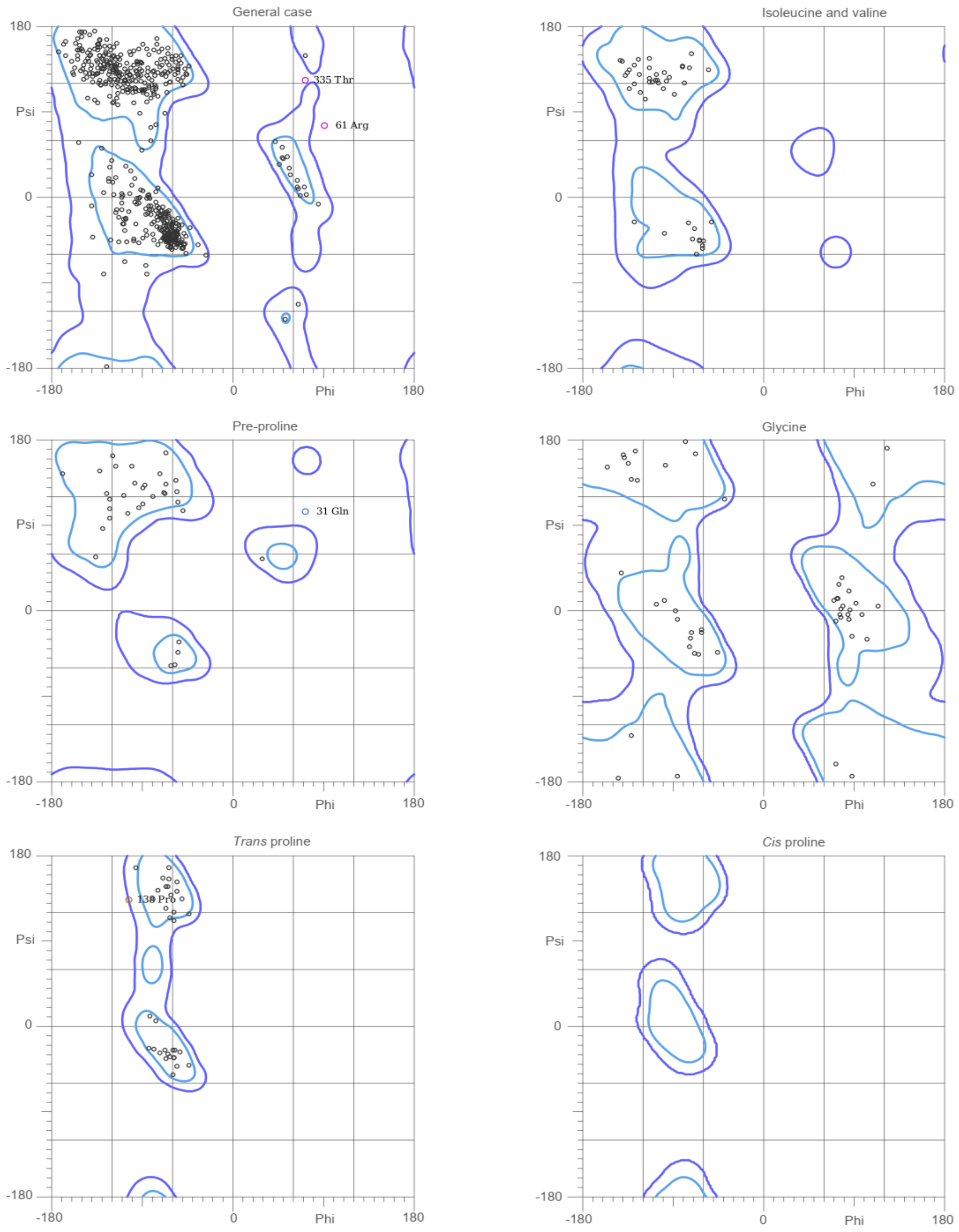

96.0% (646/673) of all residues were in favored (98%) regions.
99.4% (669/673) of all residues were in allowed (>99.8%) regions.

There were 4 outliers (phi, psi):
31 Gln (73.0, 105.3)
61 Arg (91.1, 76.3)
138 Pro (-104.0, 134.9)
335 Thr (72.0, 124.1)

(A) *Serratia marcescens* OPB

Supplementary Materials

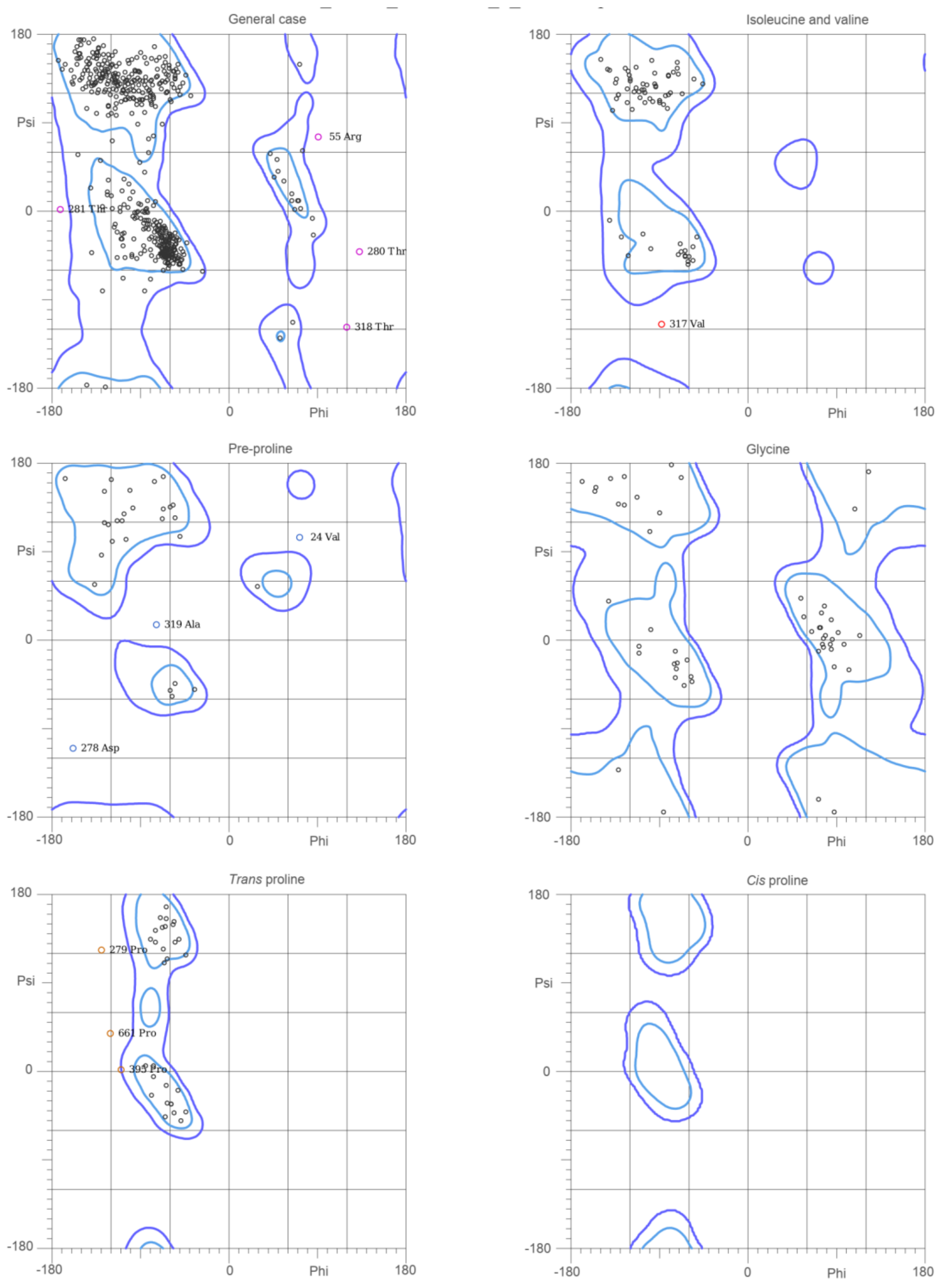

94.4% (637/675) of all residues were in favored (98%) regions.
98.4% (664/675) of all residues were in allowed (>99.8%) regions.

There were 11 outliers (phi, psi):

24 Val (73.0, 105.3)
55 Arg (91.1, 76.3)
278 Asp (-159.2, -110.9)
279 Pro (-130.6, 124.9)
280 Thr (133.4, -41.3)
281 Thr (-172.7, 3.0)
317 Val (-89.0, -115.0)
318 Thr (120.2, -118.2)
319 Ala (-74.1, 16.5)
395 Pro (-111.0, 2.7)
661 Pro (-121.0, 39.9)

(B) *Stenotrophomonas maltophilia* OPB

Supplementary Materials

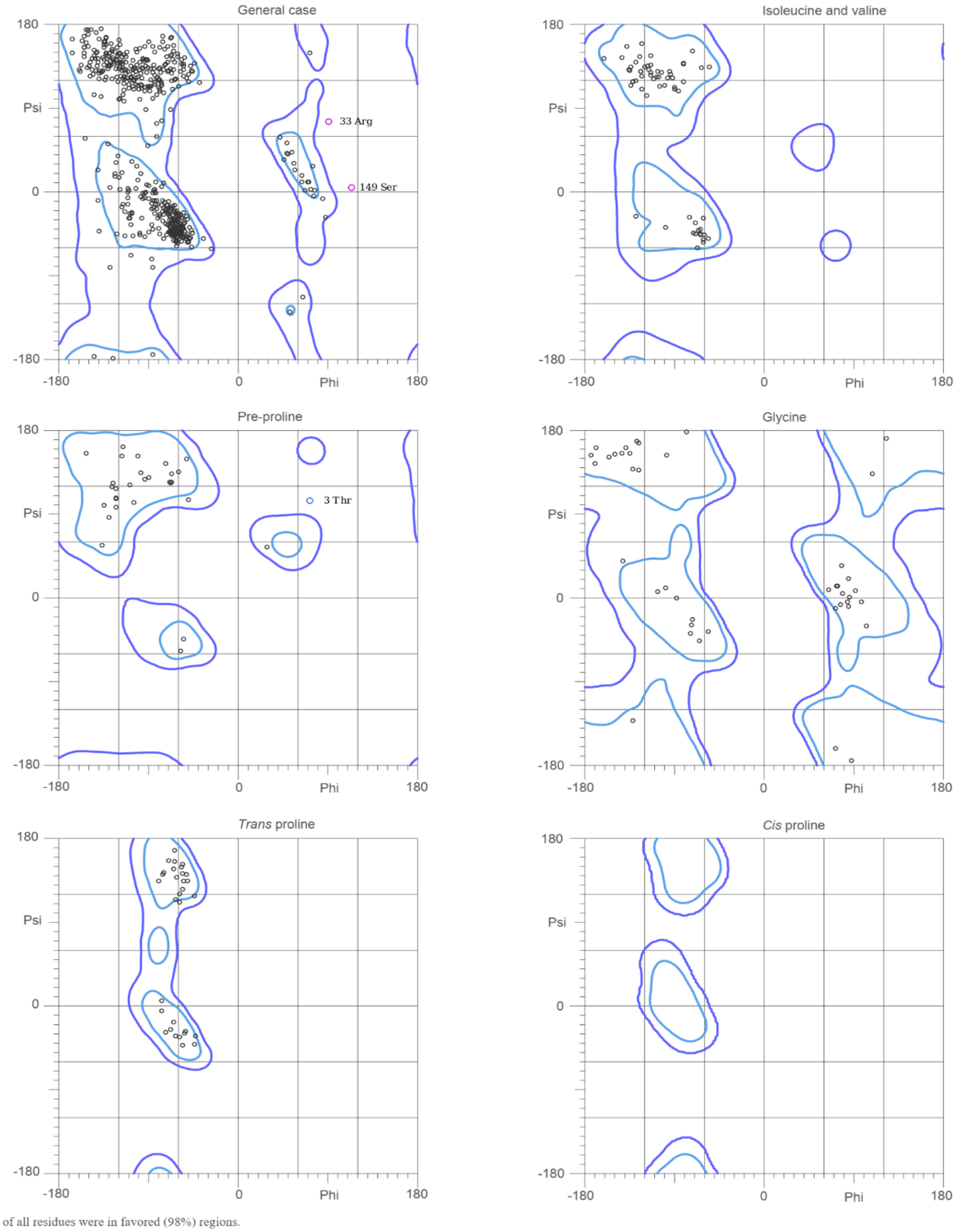

96.1% (648/674) of all residues were in favored (98%) regions.
99.6% (671/674) of all residues were in allowed (>99.8%) regions.

There were 3 outliers (phi, psi):
3 Thr (73.0, 105.3)
33 Arg (91.1, 76.3)
149 Ser (114.9, 5.6)

(C) *Serratia proteamaculans* OPB

Figure S2: Ramachandran plots of OPB targets and template showing φ and ψ torsional angle distributions: (A) *Serratia marcescens*, (B) *Stenotrophomonas maltophilia*, (C) *Serratia proteamaculans*

Supplementary Materials

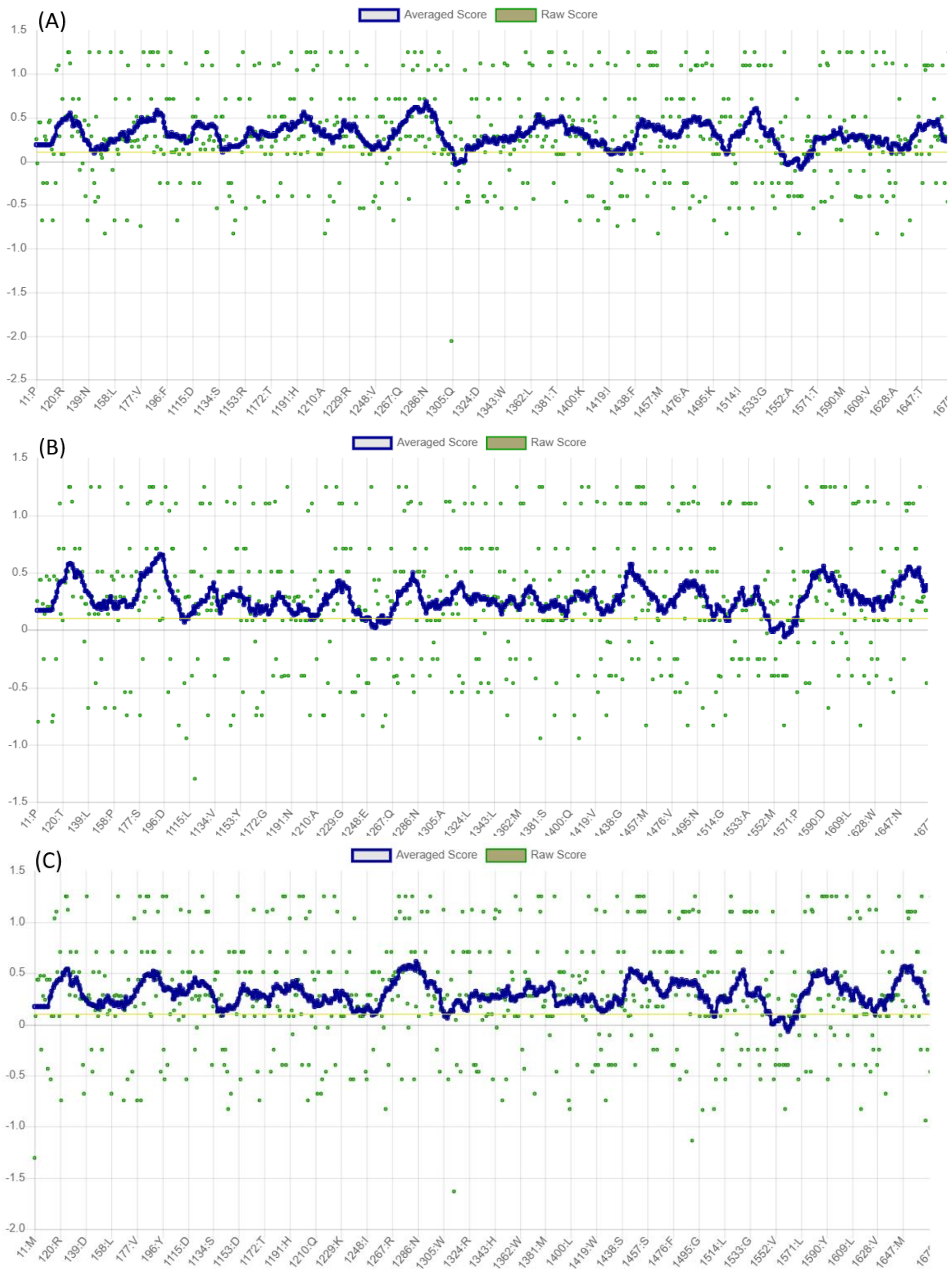

Figure S3: Verify3D structural validation profiles for OPBs, with ≥80% of residues scoring ≥0.1: (A) *S. marcescens*, (B) *S. maltophilia*, (C) *S. proteamaculans*

Supplementary Materials

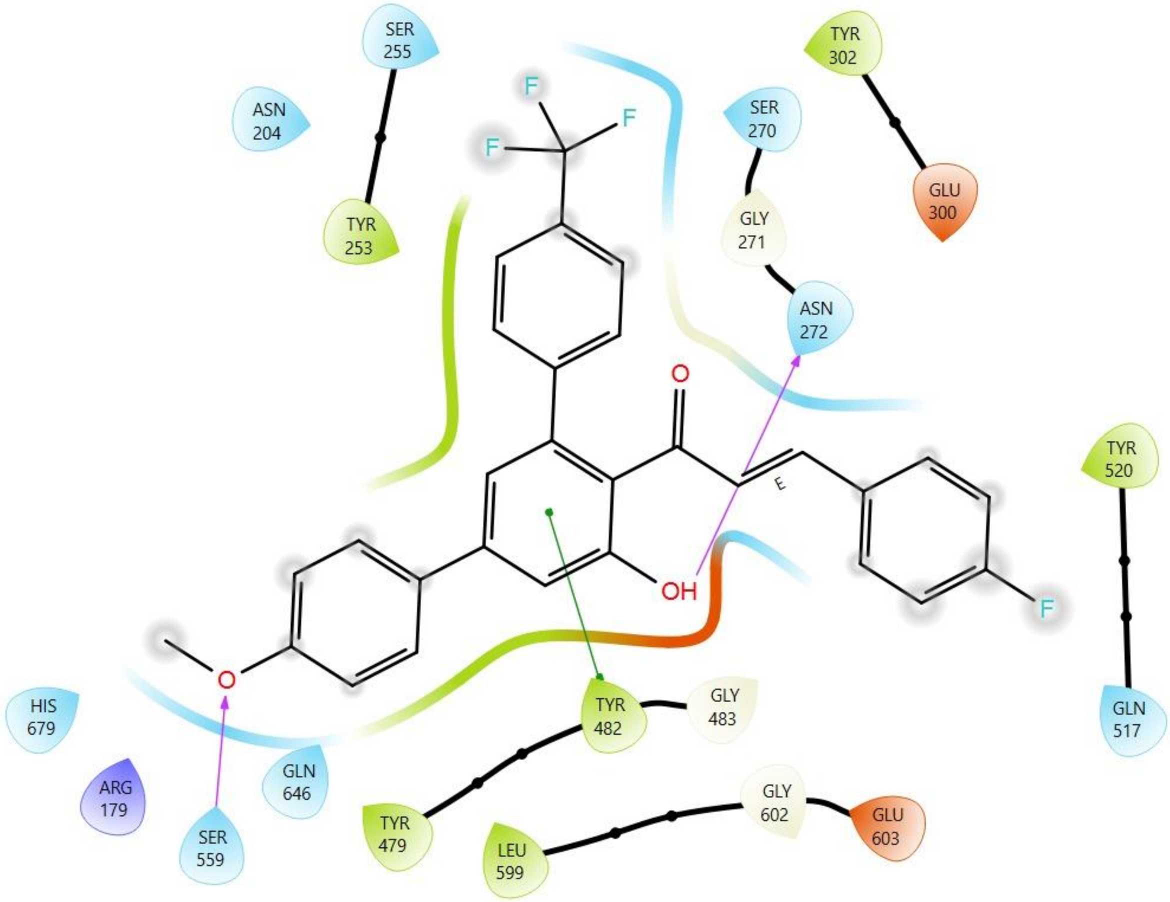

Figure S4: 2D interaction map of compound C-132 within the catalytic pocket of *Serratia marcescens* oligopeptidase B (OPB), highlighting key binding interactions.

Supplementary Materials

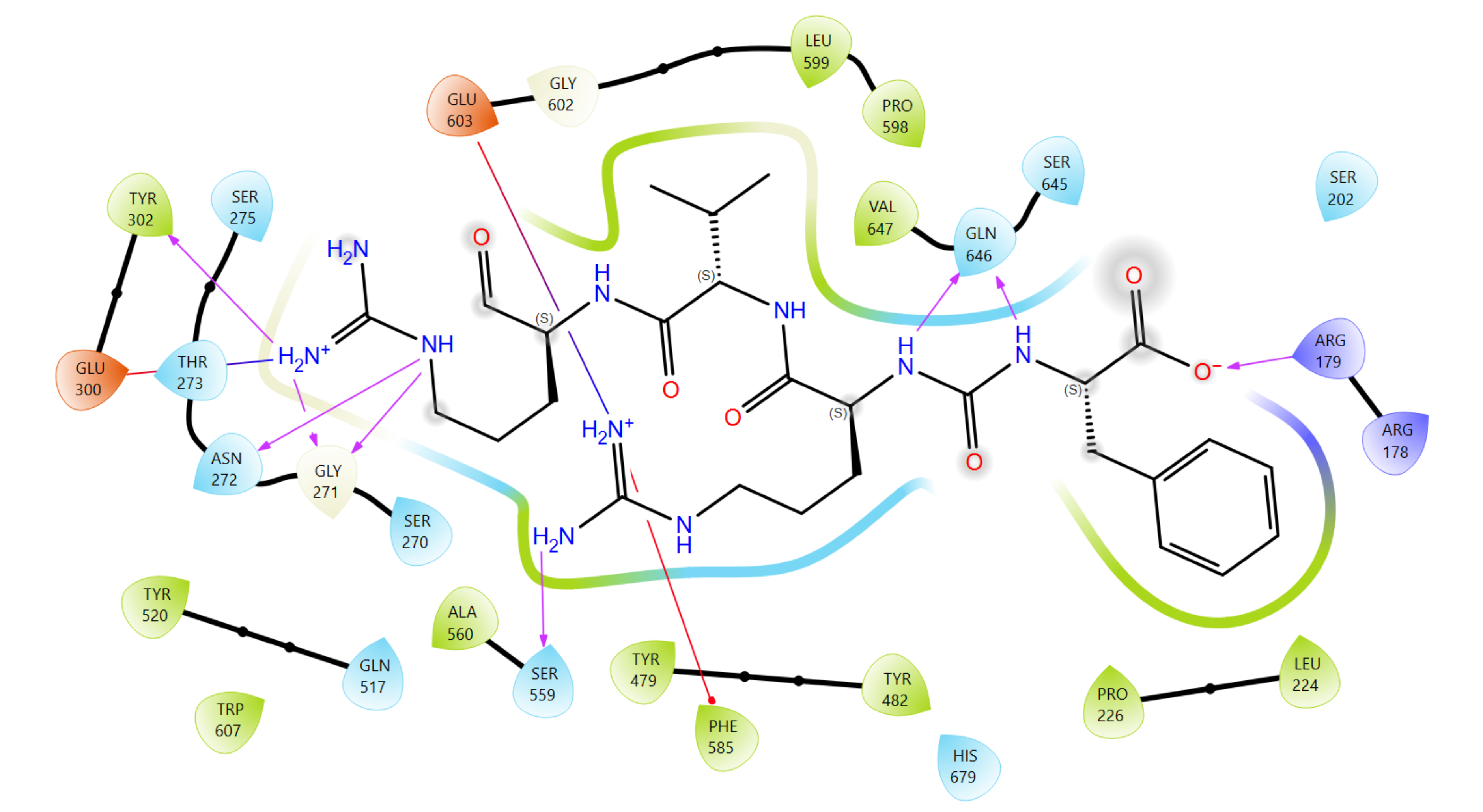

Antipain

Supplementary Materials

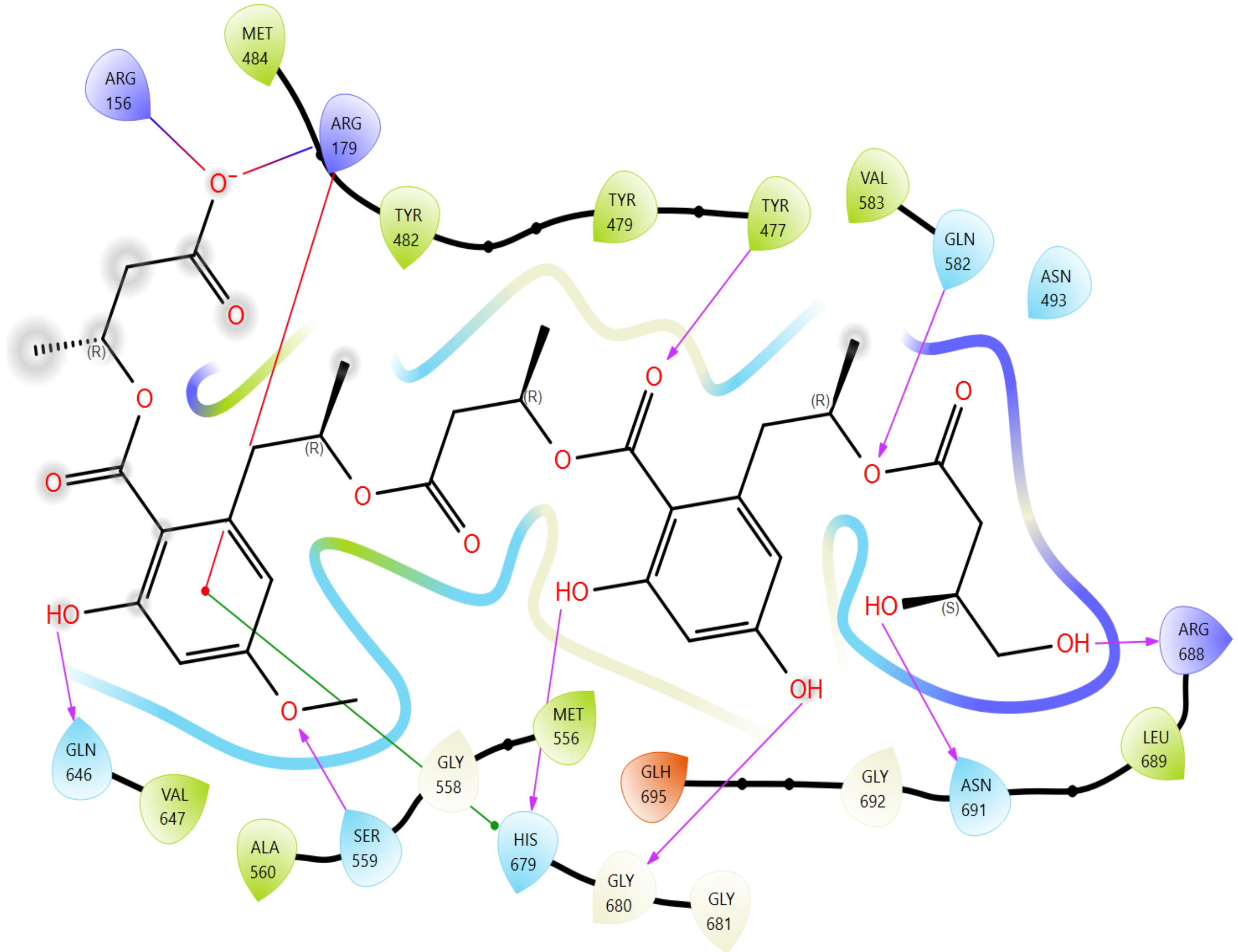

Calcaride D

Supplementary Materials

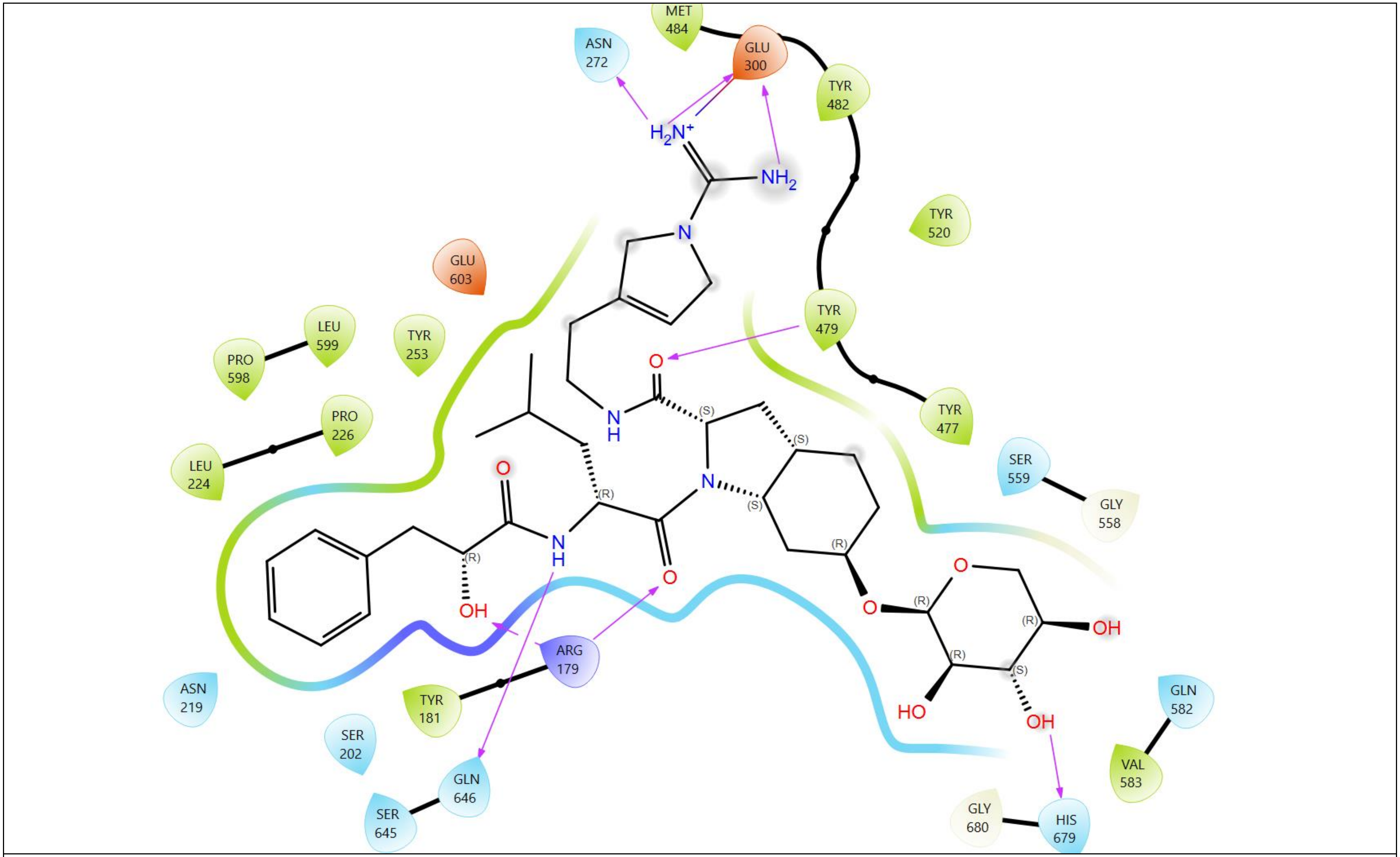

Aeruginoside 126A

Supplementary Materials

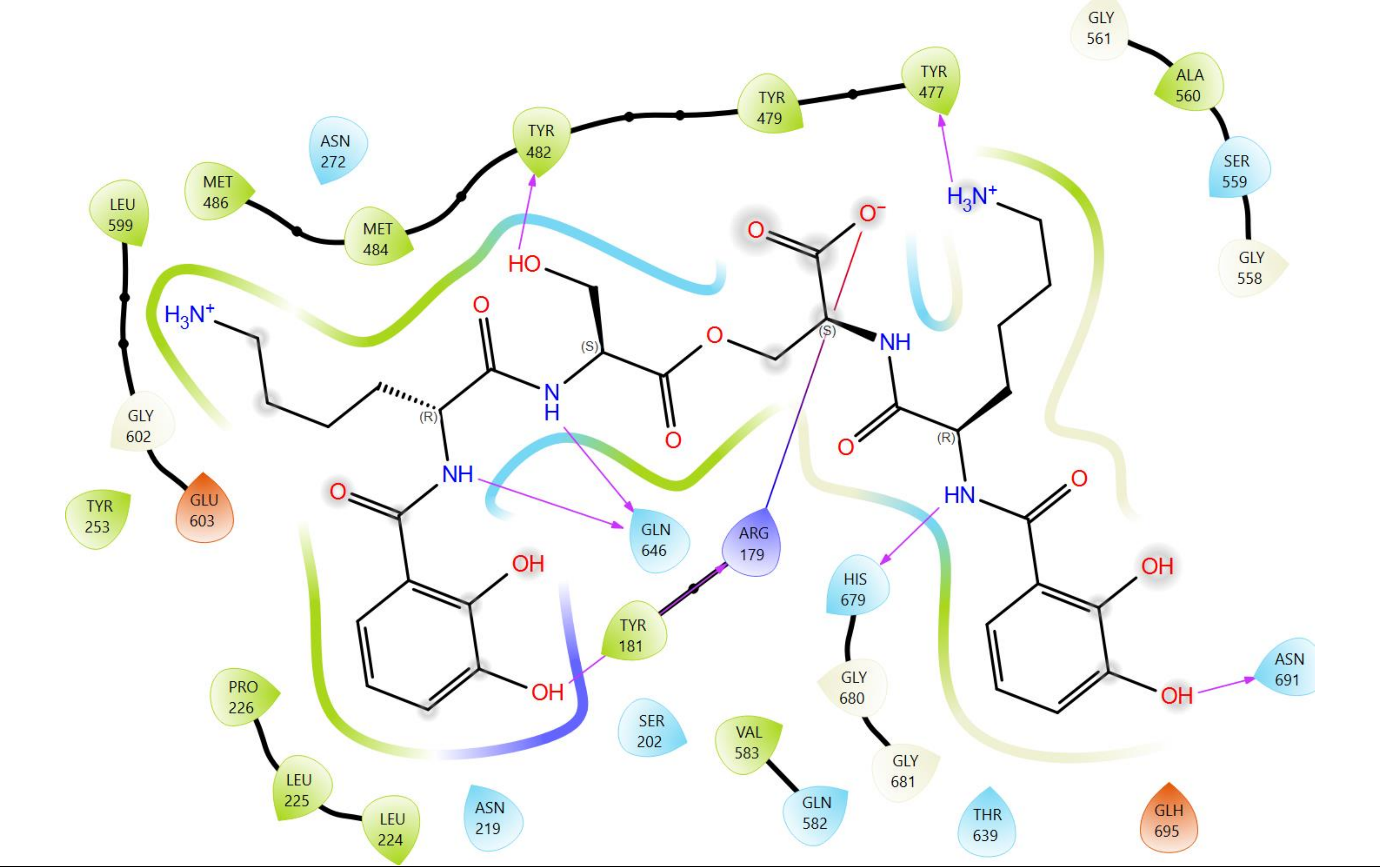

Dichrysobactin

Supplementary Materials

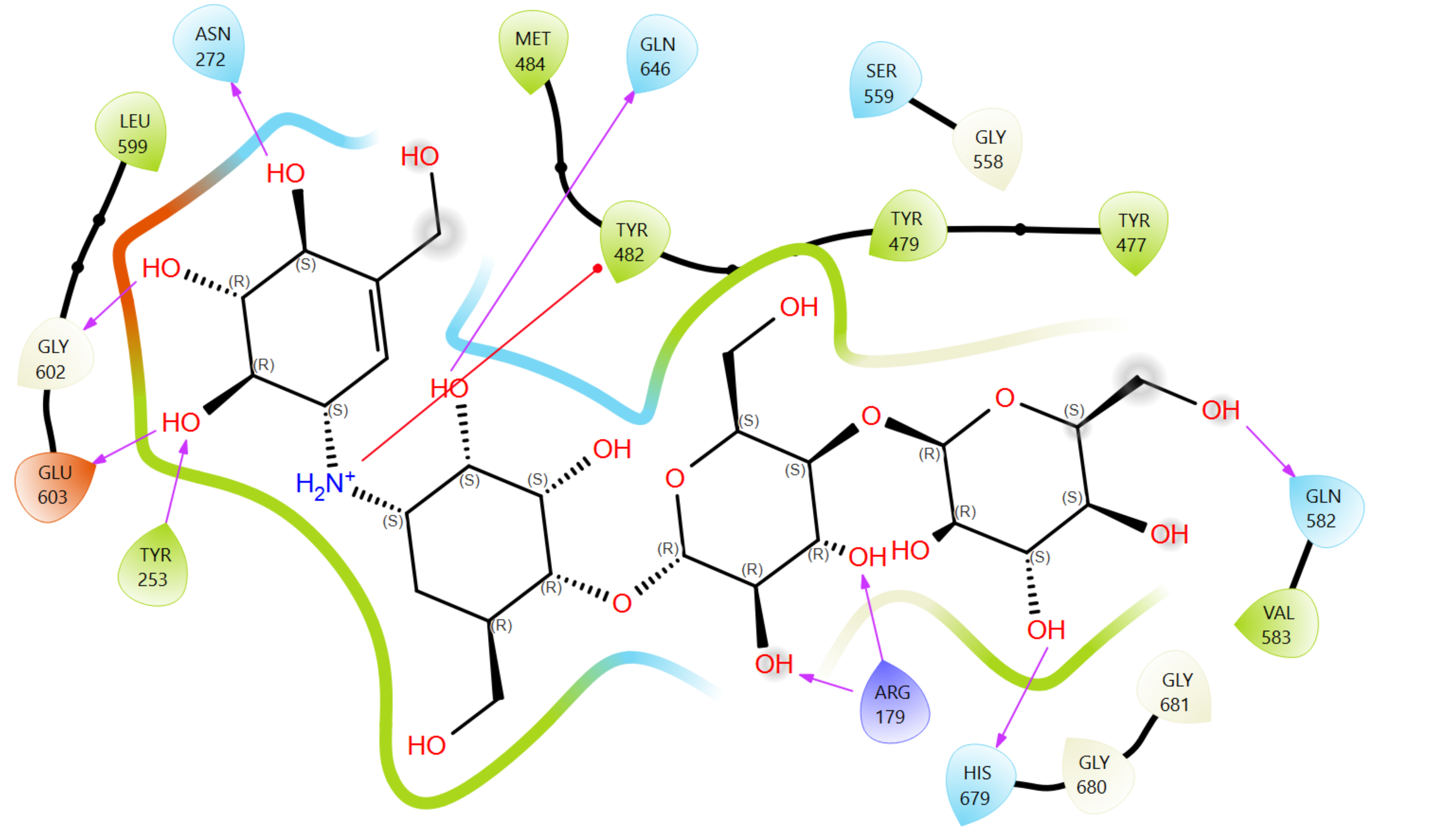

Validamycin E

Supplementary Materials

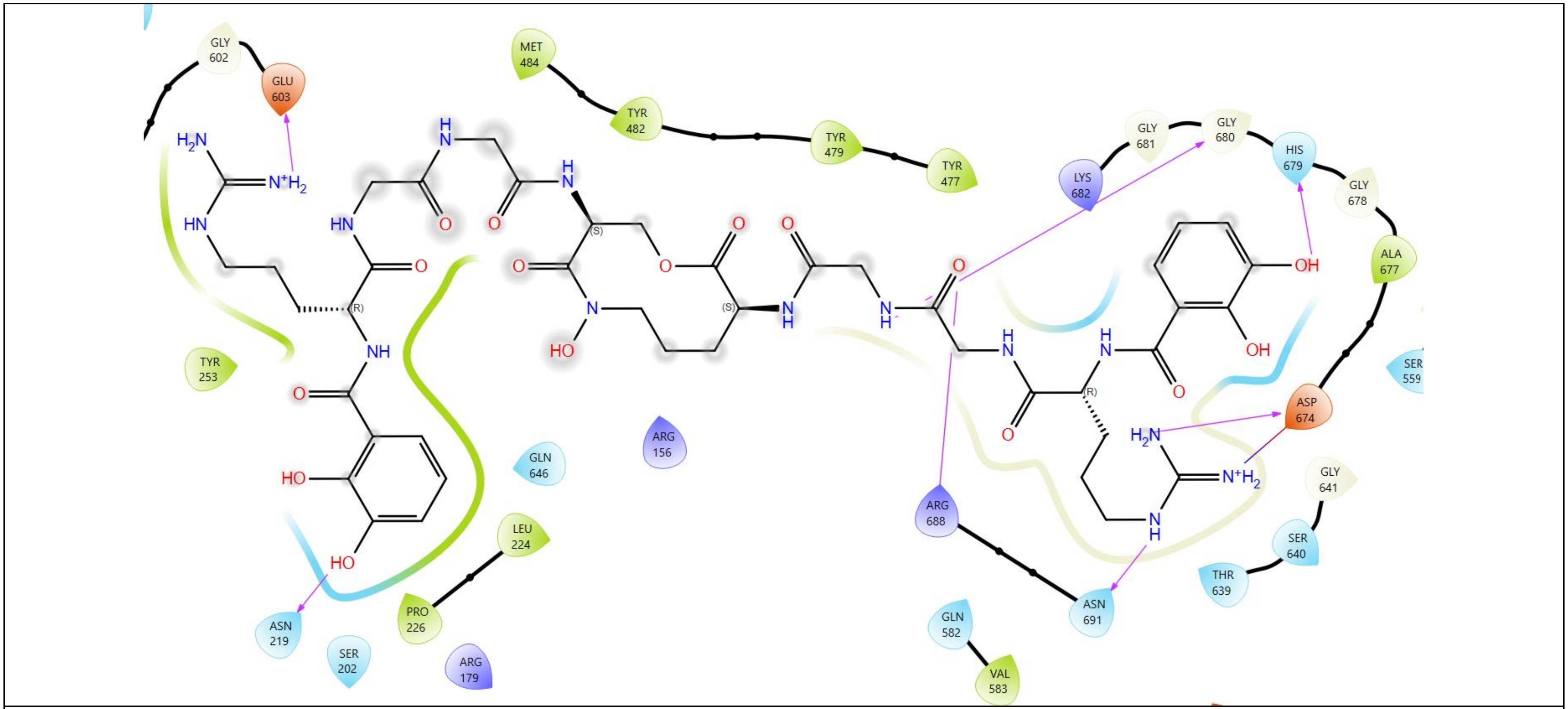

Fuscachelin A

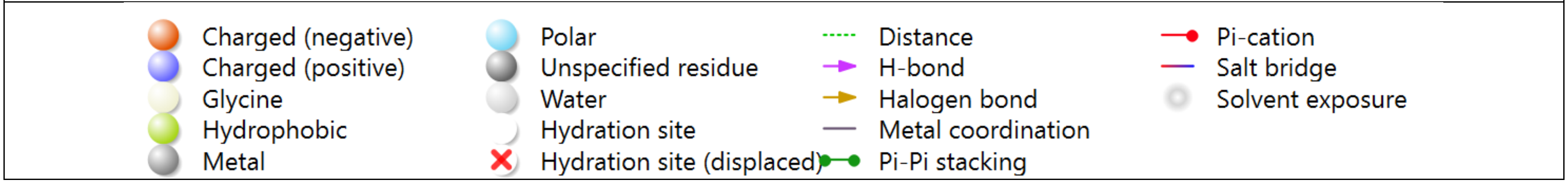

Figure S5: 2D interaction map of the top five inhibitors (Calcaride D, Aeruginoside 126A, Dichrysobactin, Validamycin E, and Fuscachelin A) along with the reference inhibitor Antipain, highlighting their binding interactions with the catalytic site of *Serratia marcescens* OPB.

Supplementary Materials

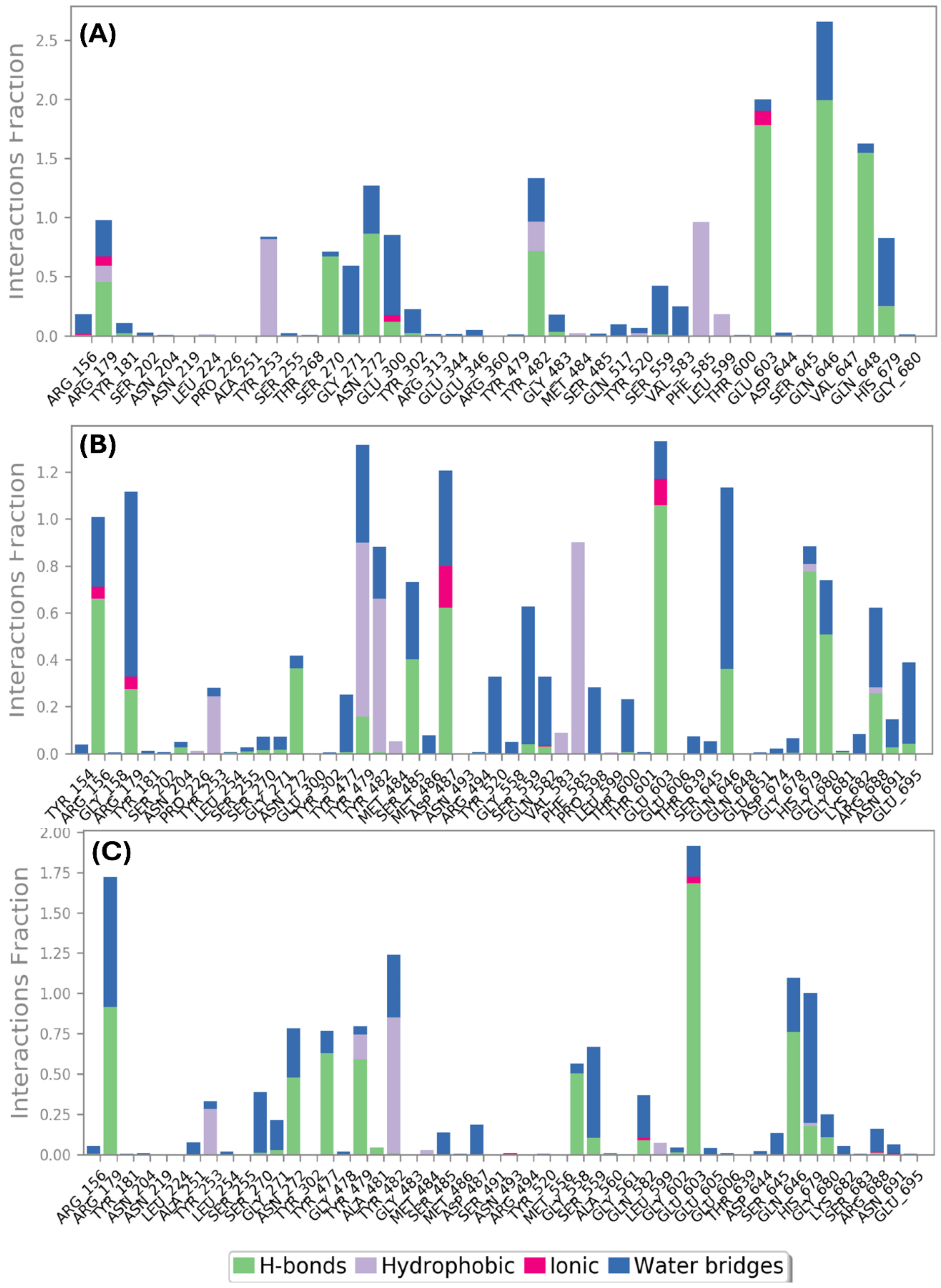

Figure S6: Histogram representation of the interactions between Antipain (A), Dichrysoisobactin (B), and Validamycin E (C) with *S. marcescens* OPB throughout the simulation run.

Supplementary Materials

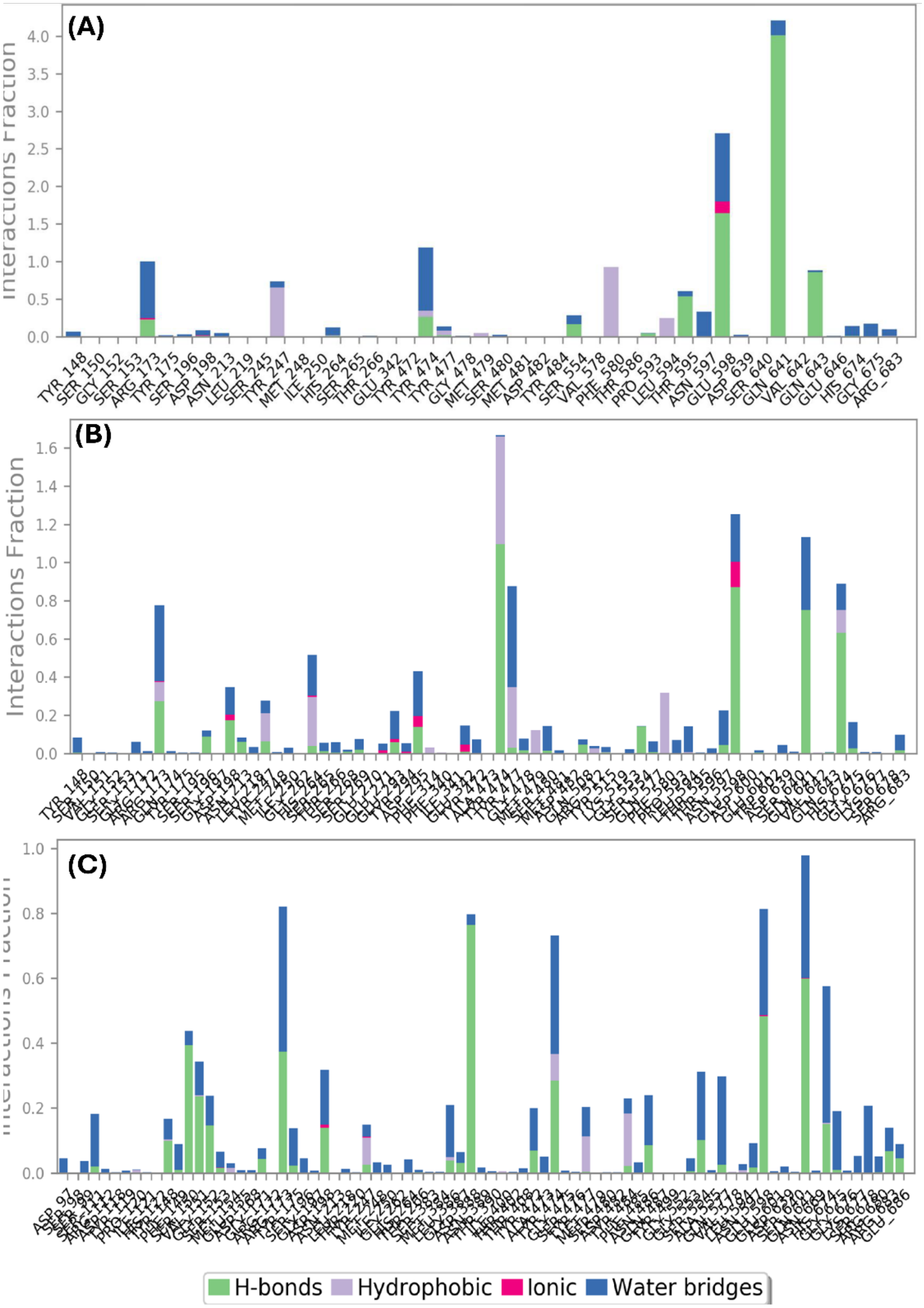

Figure S7: Histogram representation of the interactions between Antipain (A), Dichrysoisobactin (B), and Validamycin E (C) with *S. maltophilia* OPB throughout the simulation run.

Supplementary Materials

Table S1: Physicochemical and pharmacokinetics descriptors of the identified hits calculated with Qikprop

Property	Description	Permissible range	Antipain	Dichrysobactin	Validamycin E
#stars	Number of property or descriptor values that fall outside the 95% range of similar values for known drugs.	(0–5)	11	11	13
#amide	Number of non-conjugated amide groups	(0–1)	3	2	0
#rotor	Number of non-trivial, non-hindered rotatable bonds	(0–15)	21	28	24
#rtvFG	Number of reactive functional groups	(0–2)	0.00	1	2
mol_MW	Molecular weight of the molecule.	(130–725)	604.70	720.73	659.63
SASA	Total solvent accessible surface area	(300–1000)	1052.84	1150.15	885.86
FOSA	Hydrophobic component of the SASA	(0–750)	420.31	350.64	409.62
FISA	Hydrophilic component of the SASA	(7–330)	464.37	545.15	467.86
PISA	π (carbon and attached hydrogen) component of the SASA	(0–450)	168.16	254.35	8.37
WPSA	Weakly polar component of the SASA	(0–175)	0.00	0.00	0.00
donorHB	Estimated number of hydrogen bonds that would be donated by the solute to water molecules in an aqueous solution.	(0–6)	10	8	15
accptHB	Estimated number of hydrogen bonds that would be accepted by the solute from water molecules in an aqueous solution	(2–20)	12	15	32
QPlogPo/w	Predicted octanol/water partition coefficient.	(−2–6.5)	0.27	-1.95	-7.18
QPPCaco	Predicted apparent Caco-2 cell permeability in nm/sec	(<25poor, >500 great)	0.04	0.001	0.09
QPlogS	Predicted aqueous solubility	–6.5 – 0.5	-4.46	-3.25	0.92
QPlogBB	Predicted brain/blood partition coefficient.	–3.0 – 1.2	-6.80	-7.86	-5.58
QPPMDCK	Predicted apparent MDCK cell permeability in nm/sec.	<25 poor >500 great	0.03	0.00	0.02
#metab	Number of likely metabolic reactions.	(1 – 8)	9	13	15
QPlogKhsa	Prediction of binding to human serum albumin.	−1.5–1.5	-1.28	-1.06	-2.69
QPlogHERG	Predicted IC_{50} value for blockage of HERG K^+ channels.	Concern below –5	-1.05	-4.59	-5.83
Human Oral Absorption	Predicted qualitative human oral absorption	1, 2, or 3 for low, medium, or high.	1	1	1
%Human Oral Absorption	Predicted human oral absorption on 0 to 100% scale.	>80% is high	0.00	0.00	0.00
PSA	VdW surface area of polar nitrogen and oxygen atoms	7–200	313.94	357.02	299.04
Rule Of Five	Number of violations of Lipinski's rule of five	maximum is 4	3	3	3
Rule Of Three	Number of violations of Jorgensen's rule of three.	maximum is 3	2	2	2
